# Brazilian Society of Otology Task Force – bone-anchored hearing system – recommendations based on strength of evidence

**DOI:** 10.1016/j.bjorl.2026.101854

**Published:** 2026-07-22

**Authors:** Rogério Hamerschmidt, Felippe Felix, Robinson Koji Tsuji, Mariana Moreira de Castro Denaro, Pauliana Lamounier, Arthur Menino Castilho, Vagner Antonio Rodrigues Silva

**Affiliations:** a Universidade Federal do Paraná, Department of ENT, Curitiba, PR, Brazil; b Universidade Federal do Rio de Janeiro (UFRJ), Hospital Universitário Clementino Fraga Filho (HUCFF), Rio de Janeiro, RJ, Brazil; c Universidade de Sao Paulo (USP), Faculdade de Medicina, Department of Otolaryngology, São Paulo, SP, Brazil; d Universidade Federal de Minas Gerais (UFMG), Hospital das Clínicas, Belo Horizonte, MG, Brazil; e Department of Otorhinolaryngology of CRER ‒ Centro de Reabilitação e Readaptação Dr Henrique Santillo, Goiânia, GO, Brazil; f Universidade Estadual de Campinas (Unicamp), Faculdade de Ciências Médicas (FCM), Department of Otolaryngology, Head and Neck Surgery, Campinas, SP, Brazil

**Keywords:** Bone Conduction Hearing Aids, Hearing Loss, Conductive, Hearing Loss, Mixed, Hearing Loss, Unilateral, Single-Sided Deafness, Osseointegration, Implantable Hearing Devices, Microtia, Congenital Abnormalities, Otitis Media, Chronic, Cholesteatoma, Tinnitus, Cochlear Nerve Diseases, Evidence-Based Medicine

## Abstract

**Objectives:**

To provide evidence-based recommendations for the indication of bone anchored hearing system in adults and children.

**Methods:**

Task force members were educated on knowledge synthesis methods, including electronic database search, review and selection of relevant citations, and critical appraisal of selected studies. Articles written in English or Portuguese were eligible for inclusion. The American College of Physicians’ guideline grading system and the American Thyroid Association’s guideline criteria were used for critical appraisal of evidence and recommendations for therapeutic interventions.

**Results:**

The topics were divided into four parts: 1) Non-implantable hearing devices; 2) Percutaneous systems; 3) Passive transcutaneous systems; 4) Active transcutaneous systems.

**Conclusions:**

All types of BAHSs provide significant and sustained benefits, and device selection should be individualized according to the patient’s audiological profile, anatomic considerations, and personal preferences. Active transcutaneous devices are more cost-effective for health care systems and demonstrate superior long-term safety and quality-of-life outcomes compared with percutaneous devices, but with no statistically significant difference in quality of life when compared to each other.

## Highlights

• Selection of implantable hearing technology should be based on shared decision-making with the patient/family, including discussion of benefits, limitations (including MRI), risks, self-care, and aesthetic preferences.
• Simultaneous ear reconstruction and BAHS implantation may be considered when technically feasible and safe. Staged approaches are also acceptable and may be preferred in cases of complex anatomy.
• BAHS should not be indicated as a primary treatment for tinnitus in patients with SSD and profound unilateral hearing loss.
• In otosclerosis, stapedotomy should remain the standard strategy when indicated and performed by an experienced surgeon. BAHSs/active implants should be reserved for exceptional situations (unfavorable anatomy, only-hearing ear, occupational risk).
• Passive and active transcutaneous systems should be considered preferred alternatives to percutaneous devices when the primary goal includes reducing skin complications and the need for revision surgery, accepting potential small audiometric differences.

## Introduction

Since the pioneering work of von Békésy in 1932, numerous experimental studies have demonstrated that skull vibration stimulates the inner ear in a manner comparable to air-conducted sound stimulation. This evidence was later reinforced by experiments showing similar activation of the basilar membrane via both pathways.[[1], [2], [3]] In 1965, Brånemark demonstrated that titanium implants form a strong bond with bone tissue through a process he termed osseointegration. In 1977, Tjellström inserted titanium implants into the mastoid process of 3 adult patients with conductive hearing loss and attached a vibrator to a percutaneous implant, becoming the first to use a temporal bone-anchored hearing aid.[4]

Cochlear stimulation through bone vibration occurs via 5 distinct and partially interdependent biomechanical components: 1) Sound pressure in the External Auditory Canal (EAC) resulting from vibration of the canal walls and subsequent tympano-ossicular stimulation; 2) the inertial component of the ossicular chain, due to differential motion of the ossicles relative to the skull; 3) The inertial component of cochlear fluids, in which inertial forces act directly on the cochlea, displacing the basilar membrane; 4) Compression-expansion of the otic capsule, caused by elastic deformation of the temporal bone and generation of intralabyrinthine pressure gradients; and 5) Internal transmission of intracranial pressure, potentially mediated by cerebrospinal fluid. Among these mechanisms, the inertial component of cochlear fluids is considered the primary determinant of bone conduction auditory response at clinically relevant frequencies.[2]

Bone-Anchored Hearing Systems (BAHSs) can be classified into 4 categories. The first group consists of nonsurgical systems, comprising external bone vibrators that may be coupled via headbands, elastic bands, adhesive devices, dental appliances, eyeglasses, or custom ear-canal molds, with multiple commercial models available.

Non-implantable bone conduction hearing aids have limitations in sound quality and frequency bandwidth compared with implantable BAHSs. Their main limitation is reduced high-frequency amplification, largely due to attenuation of vibrations as they pass through soft tissues. This results in lower functional gain, which may impair speech clarity, particularly in noisy environments and in patients with high-frequency hearing loss.[[5], [6], [7]]

Percutaneous systems use an abutment that penetrates the skin. They are osseointegrated into the temporal bone and allow direct coupling of the sound processor – Baha Connect (Cochlear BAS, Gothenburg, Sweden) and the Ponto system (Oticon Medical AB, Askim, Sweden). These systems have the highest amplification capacity.

Passive transcutaneous systems rely on a titanium plate osseointegrated into the temporal bone. The external processor is attached via a magnet, allowing sound transmission through intact skin – Baha Attract (Cochlear BAS, Gothenburg, Sweden) and Sophono (BHM-Tech, Grafenschachen, Austria).

Active transcutaneous systems place the transducer under the skin and temporal muscle, communicating with the external processor via radiofrequency – Bonebridge (MED-EL, Innsbruck, Austria), Osia 2 (Cochlear BAS, Gothenburg, Sweden), and Sentio 1 Min. (Oticon Medical AB, Askim, Sweden). Unlike passive systems, these devices do not require compression of the skin and soft tissues for sound transmission.

Although all BAHSs provide significant improvements in hearing thresholds and speech perception, consistent differences exist among systems. Percutaneous systems offer superior amplification, particularly at high frequencies, owing to direct transmission of vibrations to the skull without soft tissue attenuation. However, they are associated with higher rates of skin complications, need for revision surgery, and ongoing skin care.[8]

Passive transcutaneous devices are associated with good cosmetic acceptance and low complication rates but may incur a loss of up to 10‒25 dB at high frequencies due to soft tissue attenuation.[9] Active transcutaneous systems preserve skin integrity and reduce complications, and some have demonstrated hearing performance comparable to percutaneous devices in selected clinical series.[10]

The Box below compares the main systems and gains.[8,11,12]

**Table:** 

**System**	**Type**	**Bone conduction capacity (BC PTA)**
**Baha Connect** (Baha 6 Max / 7)	Percutaneous	≤55 dB HL
**Baha Connect** (Baha 5 SuperPower)	Percutaneous	≤65 dB HL
**Ponto 3** (Power / SuperPower)	Percutaneous	≤65 dB HL
**Baha Attract**	Passive transcutaneous	≤45 dB HL
**Sophono Alpha**	Passive transcutaneous	≤45 dB HL
**Bonebridge (BCI 601 / 602)**	Active transcutaneous	≤45 dB HL
**Osia 2**	Active transcutaneous	≤55 dB HL
**Sentio**	Active transcutaneous	≤45 dB HL

BCI, Bone Conduction Implant; dB HL, Decibels Hearing Level; PTA, Pure-Tone Average.

The aim of this study is to provide evidence-based recommendations for the indication of BAHSs in adults and children.

## Methods

The Brazilian Society of Otology (Sociedade Brasileira de Otologia, SBO) met some members to discuss the topic of this guideline. Each author was asked to write a text with the current literature on the topic, based on evidence and containing the elements discussed during the meeting. A rapporteur prepared the final text, which was reviewed by additional coauthors and the Brazilian Journal of Otorhinolaryngology editor.

This guideline is not intended to be a substitute for individual professional judgment. Physicians should always act and decide in a way that they believe is best for their patients, regardless of guideline recommendations. They should also operate within their scope of practice and in accordance with their training. The guidelines represent the best judgment of a team of experienced physicians addressing the scientific evidence for a given topic.

The grading system of the American College of Physicians (ACP) was used in this guideline, relating to critical appraisal and recommendations on therapeutic interventions (Table 1, Table 2).[13] An important component of this guideline was judged to be critical appraisal of diagnostic testing studies. However, the ACP guideline grading system was not designed for this purpose.[[14], [15], [16]]

**Table 1:** Interpretation of the American College of Physicians’ Guideline Grading System (for therapeutic interventions).

Recommendation	Clarity of risk/benefit	Implications
Strong recommendation	Benefits clearly outweigh harms and burdens, or vice versa.	*Patients*: Most would want course of action; a person should request discussion if an intervention is not offered.
		*Clinicians*: Most patients should receive the recommended course of action.
		*Policymakers*: The recommendation can be adopted as policy in most circumstances.
Weak recommendation	Benefits closely balanced with harms and burdens.	*Patients*: Many would want course of action, but some may not; the decision may depend on individual circumstances.
		*Clinicians*: Different choices will be appropriate for different patients; the management decision should be consistent with patients’ preferences and circumstances.
		*Policymakers*: Policymaking will require careful consideration and stakeholder input.
No recommendation	Balance of benefits and risks cannot be determined.	Decisions based on evidence cannot be made.

**Table 2:** Recommendations (for therapeutic interventions) based on strength of evidence.

Recommendation and evidence of quality	Description of supporting evidence^a^	Interpretation
Strong recommendation		
High-quality evidence	RCT without important limitations or overwhelming evidence from observational studies	Can apply to most patients in most circumstances without reservation
Moderate-quality evidence	RCT with important limitations or strong evidence from observational studies	Can apply to most patients in most circumstances without reservation
Low-quality evidence	Observational studies/case studies	May change when higher-quality evidence becomes available
Weak recommendation		
High-quality evidence	RCT without important limitations or overwhelming evidence from observational studies	Best action may differ based on circumstances or patients’ values
Moderate-quality evidence	RCT with important limitations or strong evidence from observational studies	Best action may differ based on circumstances or patients’ values
Low-quality evidence	Observational studies/case studies	Other alternatives may be equally reasonable
Insufficient	Evidence is conflicting, of poor quality, or lacking	Insufficient evidence to recommend for or against

a This description of supporting evidence refers to therapy, therapeutic strategy, or prevention studies. The description of supporting evidence is different for diagnostic accuracy studies. RCT, multicenter randomized controlled trial.

The American Thyroid Association (ATA) created a diagnostic test appraisal system that used the following methodological elements: consecutive recruitment of patients representative of clinical practice, use of an appropriate reference gold standard, directness of evidence (target population of interest, testing procedures representative of clinical practice, and relevant outcomes), precision of diagnostic accuracy measures (confidence intervals for estimates such as sensitivity and specificity), and consistency of results across studies using the same test that was also used in this guideline[15] (Table 3, Table 4).

**Table 3:** Interpretation of the American Thyroid Association Guideline for Diagnostic Tests.

Recommendation	Accuracy of diagnostic information versus risks and burden of testing	Implications
Strong recommendation	Knowledge of the diagnostic test result clearly outweighs risks and burden of testing or vice versa.	*Patients*: In the case of an accurate test for which benefits outweigh risks/burden, most would want the diagnostic to be offered (with appropriate counseling). A patient should request discussion of the test if it is not offered. In contrast, for a test in which risks and burden outweigh the benefits, most patients should not expect the test to be offered.
		*Clinicians*: In the case of an accurate test for which benefits outweigh risks/burden, most patients should be offered the diagnostic test (and provided relevant counseling). Counseling about the test should include a discussion of the risks, benefits, and uncertainties related to testing (as applicable), as well as the implications of the test result. In contrast, for a test in which risks and burden outweigh the perceived benefits, most patients should not be offered the test, or if the test is discussed, the rationale against the test should, for the particular clinical situation, be explained.
		*Policymakers*: In the case of an accurate test for which benefits outweigh risks/burden, availability of the diagnostic test should be adopted in health policy. In contrast, for a test in which risks and burden outweigh the perceived benefits, some restrictions on circumstances for test use may need to be considered.
Weak recommendation	Knowledge of the diagnostic test result is closely balanced with risks and burden of testing.	*Patients*: Most would want to be informed about the diagnostic test, but some would not want to seriously consider undergoing the test; a decision may depend on the individual circumstances (e.g., risk of disease, comorbidities, or other), the practice environment, feasibility of optimal execution of the test, and consideration of other available options.
		*Clinicians*: Different choices will be appropriate for different patients, and counseling about the test (if being considered) should include a discussion of the risks, benefits, and uncertainties related to testing (as applicable), as well as the implications of the test result. The decision to perform the test should include consideration of the patients’ values, preferences, feasibility, and the specific circumstances. Counseling the patient on why the test may be helpful or not, in her/his specific circumstance, may be highly valuable in the decision-making process.
		*Policymakers*: Policymaking decisions on the availability of the test will require discussion and stakeholder involvement
No recommendation	Balance of knowledge of the diagnostic test result cannot be determined.	Decisions on the use of the test based on evidence from scientific studies cannot be made.

**Table 4:** Recommendations (for diagnostic interventions) based on strength of evidence.

Recommendation and evidence of quality	Methodological quality of supporting evidence	Interpretation
Strong recommendation		
High-quality evidence	Evidence from one or more well-designed nonrandomized diagnostic accuracy studies (i.e., observational ‒ cross-sectional or cohort) or systematic reviews/meta-analyses of such observational studies (with no concern about internal validity or external generalizability of the results)	Implies the test can be offered to most patients in most applicable circumstances
Moderate-quality evidence	Evidence from nonrandomized diagnostic accuracy studies (cross-sectional or cohort), with one or more possible limitations causing minor concern about internal validity or external generalizability of the results	Implies the test can be offered to most patients in most applicable circumstances without reservation
Low-quality evidence	Evidence from nonrandomized diagnostic accuracy studies with one or more important limitations causing serious concern about internal validity or external generalizability of the results	Implies the test can be offered to most patients in most applicable circumstances, but the utilization of the test may change when higher-quality evidence becomes available
Weak recommendation		
High-quality evidence	Evidence from one or more well-designed nonrandomized diagnostic accuracy studies (i.e., observational ‒ cross-sectional or cohort) or systematic reviews/meta-analyses of such observational studies (with no concern about internal validity or external generalizability of the results)	The degree to which the diagnostic test is seriously considered may differ depending on circumstances or patients’ or societal values
Moderate-quality evidence	Evidence from nonrandomized diagnostic accuracy studies (cross-sectional or cohort), with one or more possible limitations causing minor concern about internal validity or external generalizability of the results	The degree to which the diagnostic test is seriously considered may differ depending on individual patients’/practice circumstances or patients’ or societal values
Low-quality evidence	Evidence from nonrandomized diagnostic accuracy studies with one or more important limitations causing serious concern about internal validity or external generalizability of the results	Alternative options may be equally reasonable.
Insufficient	Evidence may be of such poor quality, conflicting, lacking (i.e., studies not done), or not externally generalizable to the target clinical population such that the estimate of the true effect of the test is uncertain and does not permit a reasonable conclusion to be made.	Insufficient evidence exists to recommend for or against routinely offering the diagnostic test.

## Results

Indication for BAHSs requires a comprehensive audiologic evaluation. The cornerstone examination is pure-tone audiometry (air-conduction and bone-conduction) to characterize the type and degree of hearing loss. The classic indication includes conductive or mixed hearing loss with bone-conduction thresholds ≤55 Db HL at 500, 1000, 2000, and 3000 Hz,[[17], [18], [19]] in patients who derive insufficient benefit from conventional hearing aids, as well as in those with unilateral sensorineural hearing loss (Single-Sided Deafness, SSD).[18,19]

Because BAHSs transmit sound through skull bone conduction, they are capable of stimulating both cochleae even when fitted unilaterally. Patients with symmetric bilateral conductive or mixed hearing loss (interaural difference in average bone-conduction thresholds at 0.5, 1, 2, and 3 kHz should not exceed 10 dB and should be less than 15 dB at all frequencies) may be candidates for bilateral fitting.[20] In asymmetric conductive or mixed hearing loss, implantation is generally preferred on the side with better bone-conduction thresholds. In uncooperative patients, Auditory Brainstem Response/Cortical Auditory Evoked Potentials (ABR/CAEPs) may be used to estimate hearing thresholds with Baha stimulation.[21]

Speech discrimination plays a central role in evaluating candidates for all hearing devices. Although clinically relevant, speech discrimination lacks consistent standardization in the BAHS literature. Patients with conductive or mixed hearing loss are expected to demonstrate good sound discrimination. Most studies prioritize free-field tonal gains and **hearing thresholds**. Speech metrics are often reported as secondary, protocol-dependent, and heterogeneous, limiting reliable direct comparisons between available technologies.[[22], [23], [24]]

High-Resolution Computed Tomography (HRCT) of the temporal bone is important to assess local anatomy, bone thickness, the position of the middle and posterior fossa dura and of the sigmoid sinus, and middle ear structures, ensuring surgical feasibility and safety.[25] A minimum temporal bone thickness of ≥ 3 mm at the implant site is recommended, particularly for percutaneous systems. HRCT is essential for planning, especially in pediatric patients and those with craniofacial anomalies.[26,27]

A preoperative trial with a non-implantable device (elastic band, headband, or adhesive adapter) is indispensable and should ideally be conducted over a few days to allow patients to experience the anticipated benefit of the surgery. Surgeons may naturally become enthusiastic about the potential benefits of the procedure, and this enthusiasm can inadvertently influence patient expectations. In this context, a preoperative trial becomes an irreplaceable tool for an objective assessment of the case, reducing the risk of treatment failure due to patient dissatisfaction.

Maximum Power Output (MPO) is a key parameter in assessing the functional effectiveness of bone-conduction devices, as it defines the upper limit of stable and clinically usable cochlear stimulation.[2,28,29] MPO reflects the overall biomechanical capacity of the system, resulting from the interaction between the transducer, the mechanical coupling to the temporal bone, and the physical limits of vibration production.[1,2,28] It establishes the maximum dynamic range potentially available for auditory signal processing in each patient.[30]

Hearing performance depends not on absolute MPO but on the relationship between the effective MPO of the system and the patient’s bone-conduction thresholds.[29,30] The effective gain perceived by the patient is typically 30‒50 dB below the technical MPO[31,32] due to 1) Skin and soft tissue attenuation (-5 to -15 dB), 2) Output compression and comfort limitations, and 3) Non-rigid coupling (particularly with Softband and SoundArc devices).[33] Critical analysis of MPO is decisive in bone-conduction device selection, especially in patients with more severe conductive or mixed losses.[23,29,30] Selecting a system whose effective MPO does not provide sufficient dynamic margin relative to bone-conduction thresholds compromises implant efficacy, regardless of signal processing sophistication.

Insufficient bone thickness, complex craniofacial malformations that preclude secure device fixation, or a history of impaired wound healing (eg, keloids and hypertrophic scars) may contraindicate surgery, particularly for percutaneous systems.[[34], [35], [36]] For active transcutaneous systems, bone integrity and adequate space to accommodate the transducer are essential for safe implantation.[37] Overall, contraindications should be assessed on an individual basis, integrating dermatologic, systemic, anatomic, and audiologic factors to ensure appropriate device selection and patient safety.

### Recommendations

I. Audiologic evaluation for BAHS candidacy should include air-conduction and bone-conduction pure-tone audiometry to characterize the type and degree of hearing loss. *(Strong recommendation – Moderate-quality evidence)*

II. In conductive or mixed hearing loss, BAHS should be considered when bone-conduction thresholds are ≤55 dB HL (500, 1000, 2000, and 3000 Hz), particularly in patients who do not benefit adequately from Personal Sound Amplification Products (PSAPs); BAHS may also be considered in selected cases of SSD. *(Strong recommendation – Moderate-quality evidence)*

III. Speech recognition testing in quiet and noise should be performed whenever possible to estimate functional benefit and guide counseling. *(Strong recommendation – Low-quality evidence)*

IV. In uncooperative candidates, ABR/CAEPs may be used to estimate thresholds and support treatment decision-making. *(Weak recommendation – Low-quality evidence)*

V. HRCT of the temporal bone should be obtained when planning implantable BAHS to assess anatomy (bone thickness, relationship to dura mater, sigmoid sinus, and middle ear) and ensure surgical safety. *(Strong recommendation – High-quality evidence)*

VI. Adequate bone thickness at the implant site must be ensured; particularly for percutaneous systems, a minimum thickness of approximately 3 mm should be respected, and alternative strategies considered if this criterion is not met. *(Strong recommendation – Low-quality evidence)*

VII. A preoperative trial with a non-implantable device (elastic band, headband, adhesive), preferably over a few days, should be performed prior to surgical indication to align expectations and reduce the risk of postoperative dissatisfaction. *(Strong recommendation – Moderate-quality evidence)*

VIII. Analysis of the MPO of the system should be incorporated into device selection, especially in more severe conductive or mixed hearing loss, to ensure an adequate dynamic margin relative to bone-conduction thresholds. *(Strong recommendation – Moderate-quality evidence)*

IX. In symmetric bilateral hearing loss, bilateral fitting may be considered (provided interaural bone-conduction differences are small); in asymmetric loss, BAHS should preferentially be placed on the side with better bone-conduction thresholds. *(Weak recommendation – Moderate-quality evidence)*

### Non-implantable hearing devices

For children younger than 5-years and for patients who are not candidates for surgery (insufficient bone thickness or clinical contraindications), the sound processor may be coupled to the skull using an elastic band, headband, eyeglass frame, ear-canal mold, maxillary bone support, or adhesive adapter. Although these devices are less efficient than surgically implanted systems due to skin and subcutaneous tissue attenuation, in addition to discomfort related to compression, caused mainly by headbands, they are widely used in clinical practice.

Nonsurgical bone-conduction hearing devices represent a well-established rehabilitation strategy for patients with conductive or mixed hearing loss and, in selected cases, with SSD. These devices differ primarily in their method of coupling to the temporal bone, which directly influences vibration transmission efficiency and the maximum achievable functional gain.[7] Available options include pressure-coupled systems – elastic bands (Baha Softband, Cochlear; Ponto Softband, Oticon), adhesive systems (ADHEAR, MED-EL), SoundArc headbands (Cochlear), and mandibular coupling devices (SoundBite).

Pressure-coupled devices demonstrate superior vibratory energy transmission to the skull, resulting in consistently greater functional gain compared with adhesive systems.[7,30] These devices require a certain degree of compression to optimize hearing gains; however, increasing skin pressure in patients using the Baha Softband has not been shown to further improve audiological outcomes.[38]

Nonsurgical bone conduction devices have gained relevance as alternatives for patients with conductive or mixed hearing loss and SSD who decline surgery or are not surgical candidates. Di Berardino et al.[39] demonstrated that non-implantable devices provide measurable hearing gains and significant improvements in speech intelligibility, including in noisy environments, compared with traditional eyeglass-mounted solutions. These findings are important as they indicate that effective bone conduction can be achieved even without rigid surgical fixation to the skull, provided that transducer positioning and coupling force are adequate to ensure effective vibration propagation.

Adhesive bone-conduction systems were introduced to eliminate continuous skin compression, thus improving comfort and long-term acceptance.[40] However, indirect coupling through an adhesive adapter limits maximum vibration transmission. Comparative studies show that although these devices significantly improve audibility and speech recognition relative to the unaided condition, functional gains tend to be lower, typically in the range of 20‒30 dB, than those achieved with pressure-coupled systems.[7]

Adhesive systems are best suited for patients with low tolerance to compression or when comfort and adherence are primary priorities, whereas headbands and elastic bands remain the nonsurgical options with the highest hearing capacity, especially in patients with greater auditory demands.

Studies have compared adhesive systems with other nonsurgical options. Favoreel et al.[40] evaluated ADHEAR versus Softband (Baha 5; Cochlear BAS, Gothenburg, Sweden) and reported a 4-frequency Pure-Tone Average (PTA4) gain of 22 dB (13.0–29.9 dB) with the ADHEAR and 23 dB (13.6–32.9 dB) with the Softband. Skarzynski et al.[7] found no statistically significant difference between ADHEAR and Baha Attract users. Kuthubutheen et al.,[41] comparing ADHEAR with a headband system, demonstrated equivalent functional gain in aided thresholds across all frequencies tested (p = 0.83).

Regarding daily usage time, Zernotti et al.[42] reported a mean daily use of 12 hours in 15 children using ADHEAR after 1-year. Dahm et al.,[43] in a randomized trial, reported a median wearing time of 8.1 hours/day for ADHEAR, significantly higher than that for Softband and Headband (4.3 hours/day), largely due to compression-related discomfort when wearing the Softband or Headband. A retrospective cohort study by Krijnen et al.,[44] involving 111 children, showed lower long-term adherence with ADHEAR, with a continued-use rate of 40.9% after a median follow-up of 24-months. Discontinuation was mainly attributed to limited perceived benefit, aesthetic concerns, and practical issues with adhesives.

SoundBite is a nonsurgical intraoral bone-conduction system consisting of an external microphone that captures sound from the impaired side, processes the signal, and wirelessly transmits it to a transducer positioned on the molar teeth. The transducer then converts the sound into mechanical vibrations that propagate through the dentition and cranial bones. This system provides hearing rehabilitation, especially for patients with unilateral hearing loss, although its use requires individualized intraoral fitting and may be associated with acoustic feedback, which is typically manageable through device adjustments. Available studies report high short-term and mid-term adherence and patient satisfaction, with more than 90% of users describing continued use and benefit at 6-months, although detailed data on daily usage duration are lacking, limiting direct comparisons with other non-implantable devices.[45,46] A key advantage of SoundBite is its superior high-frequency performance with reduced soft-tissue attenuation; however, the need for individualized intraoral fitting may limit use in some patients.[45,47] Its main limitation remains the need for intraoral fitting and initial feedback management, without a significant impact on overall satisfaction after the adaptation phase.

Nonsurgical bone-conduction devices expand the therapeutic spectrum, particularly in patients for whom age, comorbidities, dermatologic conditions, aesthetic concerns, or reluctance toward surgery limit the use of osseointegrated implants. Although audiological outcomes of non-implantable devices are generally inferior to those of active transcutaneous or percutaneous implantable systems, the balance between functional benefit, comfort, reversibility, and absence of surgical risk makes nonsurgical solutions attractive in individualized strategies. Nevertheless, significant gaps remain in the literature regarding standardized performance metrics, particularly speech discrimination, and long-term quality-of-life outcomes. Further comparative studies with larger cohorts and extended follow-up are needed to better define the role of these devices in contemporary hearing rehabilitation algorithms.

### Recommendations

X. In children aged < 5-years and in patients without anatomic or clinical conditions for surgery, nonsurgical devices should be prioritized as initial rehabilitation or as a definitive alternative when appropriate. *(Strong recommendation – Moderate-quality evidence)*

XI. When greater hearing capacity is desired, pressure-coupled systems (Softband, Headband, SoundArc) may be preferred over adhesive systems, acknowledging lower tolerance due to compression. *(Weak recommendation – Moderate-quality evidence)*

XII. Adhesive systems should be considered when comfort, pressure tolerance, and adherence are primary priorities, accepting a lower MPO. *(Weak recommendation – Moderate-quality evidence)*

XIII. Increasing pressure “beyond necessary” in Softband or Headband systems should not be pursued in expectation of consistent additional hearing gains, given the risk of discomfort and reduced adherence. *(Weak recommendation – Low-quality evidence)*

XIV. Children using adhesive systems should undergo longitudinal reassessment of benefit and adherence, given the risk of long-term discontinuation and the potential need for alternative strategies. *(Strong recommendation – Low-quality evidence)*

XV. SoundBite (intraoral system) may be considered in selected SSD cases when intraoral fitting and feedback management are acceptable, recognizing limited evidence and variable applicability. *(Weak recommendation – Low-quality evidence)*

### Percutaneous systems

Percutaneous bone-conduction hearing devices are well-established strategies for hearing rehabilitation in patients with conductive or mixed hearing loss and in those with profound SSD.[48] These systems rely on osseointegration of a titanium implant into the temporal bone, enabling direct transmission of vibratory energy to the cochlea without skin and soft tissue attenuation. They provide superior vibration efficiency compared with other bone conduction systems, particularly at mid and high frequencies.[8,24,49,50]

Surgical implantation is generally rapid and technically straightforward and represents a suitable option for patients with external ear malformations who also desire reconstruction. However, all steps of the procedure must be followed meticulously to reduce the risk of complications. Depending on patient age and cooperation, the procedure may be performed under local or general anesthesia. The abutment may be placed using either a single-stage or two-stage approach. Single-stage surgery is contraindicated in patients with poor bone quality or bone thickness ≤ 3 mm.[51]

Studies have demonstrated that percutaneous devices provide substantial functional gains, often exceeding 40 dB HL, with significant improvements in free-field thresholds and speech recognition in both quiet and noise.[19,24,28] Owing to this high vibration transmission efficiency, percutaneous systems were long regarded as the gold standard among implantable bone-conduction devices, particularly for patients with greater auditory demands and good adherence to clinical follow-up.

Percutaneous devices also play an important role in the rehabilitation of SSD, functioning as Contralateral Routing Of Signal (CROS) systems via bone conduction. Classic studies have shown improvements in sound perception, spatial awareness, and patient-reported outcomes when compared with conventional air-conduction CROS systems.[20,52,53] Although they do not restore binaural hearing, these devices represent an effective option for patients who are not candidates for cochlear implants.

The contraindications and limitations of percutaneous systems should be carefully considered. Conditions associated with a higher risk of osseointegration failure or complications include uncontrolled metabolic bone disease, prior radiotherapy to the temporal bone, immunosuppression, and limited capacity for self-care, as these systems require daily hygiene of the percutaneous site and regular follow-up.[19,36] Skin complications, such as peri-implant inflammation, local infection, and soft tissue hyperplasia, remain the principal limiting factors, although advances in surgical techniques have reduced their incidence.[35,54,55] Therefore, indication of percutaneous devices should always be individualized, balancing their superior hearing potential against local risks and patient preferences.

Both implant designs and surgical techniques have improved over time. Early approaches involved removal of subcutaneous tissue and hair follicles around the abutment. Subsequently, linear incision techniques with soft tissue preservation were introduced. Hultcrantz et al.[56] described a minimally invasive procedure for the Ponto™ BAHS (Oticon Medical), involving a 5-mm punch through the skin to remove only the tissue required to accommodate the abutment. This technique is known as Minimally Invasive Ponto Surgery (MIPS).

Current evidence indicates that MIPS does not confer a superior reduction in surgical complications compared with other percutaneous techniques, such as linear incision with tissue preservation used in the Ponto and Baha Connect systems. Randomized and cohort studies demonstrate similar rates of adverse skin reactions, inflammation, and postoperative complications between MIPS and conventional approaches, with no statistically significant differences in most complication-related outcomes.[31,57,58]

However, MIPS offers advantages in terms of shorter operative time, improved cosmetic results, and reduced postoperative skin sensitivity, despite the lack of robust evidence demonstrating a lower rate of major surgical complications or infections compared with traditional techniques.[31,57] Safety profiles are considered equivalent, and the choice of technique may be guided by factors such as patient preference, surgeon experience, and clinical context.

### Recommendations

XVI. Percutaneous systems should be considered when maximum vibration efficiency and higher functional gain are desired, provided the patient is able to perform adequate self-care and adhere to regular follow-up. *(Strong recommendation – Moderate-quality evidence)*

XVII. Single-stage surgery for percutaneous devices should be avoided in patients with poor bone quality or bone thickness ≤3 mm; a two-stage approach or alternative technology should be selected instead. *(Strong recommendation – Low-quality evidence)*

XVIII. In patients at increased risk of complications (eg, immunosuppression, prior radiotherapy, limited self-care ability, dermatoses or wound-healing disorders), percutaneous systems should be indicated with caution or avoided, favoring intact-skin alternatives when feasible. *(Strong recommendation – Low-quality evidence)*

XIX. The MIPS technique may be used for its operative and cosmetic advantages but should not be presented as superior to conventional techniques in terms of complication reduction. *(Strong recommendation – Moderate-quality evidence)*

### Passive transcutaneous systems

In passive transcutaneous BAHSs, the external sound processor is retained by a magnetic coupling system and contains the vibratory transducer responsible for converting the acoustic signal into mechanical energy. Vibration is generated over intact skin and transmitted to the implanted component through the skin, subcutaneous tissue, and muscle until it reaches the internal magnet fixed to the temporal bone. Vibration transmission efficiency depends directly on the strength of magnetic coupling, which must be sufficient to minimize energy dissipation caused by soft tissues while avoiding adverse effects related to skin compression, such as pain, ischemia, necrosis, or discontinuation of device use.

## Baha Attract

The Baha Attract system uses the same sound processors and the same osseointegrated titanium implant as the Baha Connect system. A titanium implant is placed directly into the skull in a manner similar to percutaneous BAHSs. A magnet is attached to this implant, and the skin is sutured over the assembly. After incision healing and completion of osseointegration, the external device is activated.

The processor is magnetically retained and vibrates in response to sound. Vibratory energy is transmitted through the skin and soft tissues to the internal magnet and implant, which convey the vibration to the skull. The magnetic coupling must provide adequate retention to ensure effective sound transmission while avoiding discomfort and pressure-related soft tissue complications. A soft tissue thickness of 3‒6 mm is recommended for optimal functioning of the device. Thickness greater than 6 mm increases the risk of poor magnet retention, whereas thickness less than 3 mm may result in excessive magnetic retention, potentially causing skin discomfort or injury.

## Sophono

The Sophono system (Alpha/Alpha 2) was the first transcutaneous, “abutment-free” bone-conduction system to be introduced. It is considered passive because the external processor contains the vibratory transducer and is magnetically coupled to an internal magnet fixed to the temporal bone. The system was developed to address typical complications associated with percutaneous devices while maintaining an implantable solution for conductive/mixed hearing loss and SSD rehabilitation, offering improved cosmetic acceptance and reduced daily skin care requirements.[59]

The available literature is composed predominantly of case series and retrospective studies, with a limited number of comparative analyses. In children, one study demonstrated significant improvements in PTA and Speech Recognition Threshold (SRT) with Sophono, comparable to Baha Attract in the analyzed cohort, although sample sizes were small and study design was nonrandomized.[60] In patients with conductive or mixed hearing loss, Magliulo et al.[61] evaluated the Sophono Alpha in noise conditions and reported a mean free-field gain of approximately 26.7 dB, along with improvements in SRT in quiet and in sentence recognition thresholds in noise, supporting functional efficacy despite limitations related to sample size and lack of a control group.

When compared with percutaneous systems, available data suggest that Sophono provides clinical benefit but with inherent limitations due to energy attenuation imposed by soft tissues. In a pediatric comparative study by Hol et al.,[62] both Sophono and percutaneous Baha systems produced hearing gain; however, field measurements indicated greater output with the percutaneous device. In a longer-term follow-up of children with congenital unilateral conductive hearing loss, Nelissen et al.[63] reported good tolerability of Sophono but noted that aided thresholds did not consistently reach targets below a mean PTA of 30 dB, and discontinuation of device use occurred in a subset of patients.

Regarding safety and complications, the principal concern with Sophono relates to morbidity associated with magnetic coupling: sufficient magnetic force is required for adequate performance, yet excessive compression may lead to pain, skin irritation, or pressure-related injury. A systematic review including 8 studies (86 patients, 99 implants; predominantly pediatric) reported a mean PTA improvement of approximately 31 dB and postoperative complications in about 29% of cases, most of which were minor; severe complications requiring surgical removal of the magnet occurred in approximately 3.5% of cases.[59]

### Active transcutaneous systems

In active transcutaneous hearing systems, the vibratory transducer is implanted directly into the temporal bone, typically secured by rigid osteosynthesis, while the external processor is responsible solely for capturing and processing the acoustic signal, transmitting it to the implant via radiofrequency or electromagnetic induction. Mechanical vibration is generated directly in the bone, eliminating soft tissue attenuation and obviating the need for skin compression to achieve sound transmission. Active transcutaneous systems provide greater vibration efficiency than passive systems, particularly at mid and high frequencies, and have a more favorable long-term tolerability profile.

## Bonebridge

Bonebridge (MED-EL, Innsbruck, Austria) is an active transcutaneous system in which energy is not transmitted through the skin, unlike passive systems. The Bonebridge Bone-Conduction Implant (BB-BCI) consists of 2 components: an internal implant comprising a magnet, coil, and actuator, also referred to as the Bone Conduction Floating Mass Transducer (BC-FMT), and an external sound processor.

The BB-BCI can be implanted in the mastoid, retrosigmoid, or middle fossa region. Two implant models are available: BCI-601 and BCI-602, which differ in the depth of the bone bed required to place the BC-FMT. The BCI-601 requires a deeper bone bed (8.7 mm), whereas the BCI-602 requires a shallower bed (4.5 mm). The BCI-602 system includes self-drilling screws in the implant kit. In cases in which cranial thickness is insufficient to house the implant, spacers (“lifts”) may be placed on the fixation wings of the BC-FMT.

The variability in implant positioning and the use of lifts did not result in reduced hearing gain in a study evaluating 6 cadaveric temporal bones. In each sample, a hole was drilled in each of the 3 implant locations to house the implant transducer (middle fossa, retrosigmoid, and mastoid), with and without the use of lifts of varying thickness (1‒4 mm).[64] Sound-induced vibration at the cochlear promontory was measured at 8 frequencies (0.5, 0.75, 1, 1.5, 2, 3, 4, and 6 kHz) using laser Doppler vibrometry. No significant differences were observed related to screw type, lift use, or implant location.

## Osia 2

The Osia 2 system (Cochlear™, Sydney, Australia) provides a maximum gain of 55 dB at frequencies of 0.5, 1, 2, and 4 kHz. The implant directly stimulates bone through a piezoelectric transducer. It consists of a sound processor and an internal device. Multicenter studies and case series have demonstrated low complication rates, with the most common being local infection, postoperative pain, and, rarely, the need for explantation. Most reported adverse events are mild and self-limiting, with surgical complication rates generally below 10% and explantation rates below 2% in large series.[65,66]

The primary indications for Osia 2 include conductive or mixed hearing loss with bone conduction thresholds up to approximately 55 dB HL and SSD, in both adults and children aged ≥5 years. In children, safety has also been confirmed in prospective clinical trials, leading to U.S. Food and Drug Administration approval for pediatric use.[67]

Introduced in late 2020, Osia 2 has limited data on complications. However, a meta-analysis by Key et al.,[68] including 314 patients, reported early complication rates comparable to other transcutaneous implants, with very low rates of explantation (0.11%) and surgical wound infection (1.92%). The overall mean gain was 35.0 dB Sound Pressure Level (SPL) across all types of hearing loss when compared with the unaided ear, increasing to 37.7 dB SPL in patients with conductive or mixed hearing loss.

## Sentio

The Sentio Ti implant is similar to Bonebridge. The transducer is placed into a bone bed (≤ 3 mm) created in the temporal bone and fixed with a stabilization band and 2 screws, while the implant receiver is positioned in an adjacent subperiosteal pocket. The implant does not contain an internal power source; instead, it receives energy via electromagnetic induction from a radiofrequency signal transmitted by the external Sentio 1 Min. sound processor when worn and activated.[12]

An experimental study by Ghoncheh et al.[12] evaluated the Sentio system in human cadaveric heads, comparing it with a percutaneous device (Ponto 3) implanted at the same location. Sentio demonstrated approximately 10‒15 dB lower mechanical output on average; however, repositioning the implant closer to the cochlea increased transmitted vibration by approximately 13 dB, achieving performance comparable to that of the percutaneous device at frequencies above 500 Hz. These findings highlight the critical role of optimal surgical positioning in determining the functional outcomes of active transcutaneous implants.

In a prospective multicenter study by Hol et al.,[69] the Sentio system demonstrated a favorable safety profile and clinically meaningful hearing gains. Mean free-field improvements across frequencies from 500 to 4000 Hz were approximately 30‒35 dB, with an effective functional gain of about 9 dB, reflecting efficient vibration transmission through the active transcutaneous interface. Speech recognition in quiet approached 98%, and performance in noise showed a significant improvement in Signal-to-Noise Ratio (SNR) (approximately -3 dB) compared with the preoperative condition and prior use with a softband. The absence of a randomized control group and limited follow-up underscore the need for direct comparative studies and longer-term evaluations to better define the role of the Sentio system in the bone conduction hearing rehabilitation algorithm.

Comparative studies evaluating Sentio, Bonebridge, and Osia 2 are lacking. Sentio offers a compact architecture, an integrated transducer, and greater flexibility in implant positioning, which may facilitate its use in small or sclerotic mastoids. However, the available literature remains limited and is largely derived from specialized centers or regulatory data. Independent studies with follow-up exceeding 5-years are still lacking. The consolidation of independent, long-term evidence will allow for a more precise definition of its role among available technologies and the establishment of device-specific recommendations for different patient profiles.

### Recommendations

XX. Passive transcutaneous systems may be considered when preservation of intact skin and cosmetic outcomes are a priority and the patient accepts lower MPO due to soft tissue attenuation. *(Weak recommendation – Low-quality evidence)*

XXI. In passive transcutaneous systems, soft tissue thickness and magnet strength should be carefully assessed and adjusted to balance device retention, performance, and cutaneous safety. *(Strong recommendation – Low-quality evidence)*

XXII. Users of passive systems should be monitored for pain, skin irritation, pressure-related injury, and discontinuation of device use. *(Strong recommendation – Low-quality evidence)*

XXIII. Active transcutaneous systems should be considered when preservation of intact skin is desired along with greater vibration efficiency, particularly in patients with greater auditory demands. *(Strong recommendation – Moderate-quality evidence)*

XXIV. For Bonebridge, planning of the implant site (mastoid, retrosigmoid, or middle fossa) and the use of spacers (“lifts”) should be individualized according to patient anatomy; appropriately indicated variations can maintain acceptable functional performance. *(Weak recommendation – Low-quality evidence)*

XXV. Osia 2 should be considered for conductive or mixed hearing loss with bone conduction thresholds up to approximately 55 dB HL and for SSD in adults and children aged ≥ 5-years, depending on anatomic feasibility and regulatory context. *(Strong recommendation – Moderate-quality evidence)*

XXVI. The Sentio system may be considered in experienced centers, with attention to optimization of implant positioning, while recognizing the limitations of independent, long-term evidence and the lack of robust direct comparative studies. *(Weak recommendation – Low-quality evidence)*

## Discussion

### External/Middle ear malformations

Microtia and congenital aural atresia are malformations that occur in approximately 1 in 10,000 to 20,000 live births. They are usually unilateral, with a predilection for the right ear, and are more common in boys than in girls (2.5:1). Inner ear anatomy and function are often preserved, although they may be involved in 10% to 20% of cases.[70]

Congenital malformations of the external and middle ear encompass a wide spectrum, ranging from mild auricular deformities to microtia/anotia associated with canal stenosis/atresia and middle ear anomalies. These defects arise primarily during early gestation, when the external ear (auricle + cartilaginous portion of the canal) and the osseous canal develop in a coordinated manner, explaining the frequent combined involvement of the auricle and EAC. There is marked phenotypic heterogeneity, and multiple classification systems have been proposed to guide diagnosis and reconstruction.[70,71] A substantial proportion of external ear anomalies are non-isolated and occur in association with syndromic conditions and craniofacial or systemic malformations, warranting comprehensive clinical evaluation and screening for comorbidities.[71]

Systematic reviews and clinical series indicate that 30% to 60% of patients with microtia present with extracranial anomalies or have syndromic conditions, with higher prevalence in bilateral cases, suggesting a stronger genetic basis in these scenarios. Isolated unilateral microtia is more often attributed to multifactorial or sporadic mechanisms, accounting for the lower rate of relevant genetic findings in these patients.[71,72] Genes such as TCOF1, SALL1, EYA1, SIX1, and CHD7, as well as chromosomal abnormalities detected by techniques such as array-CGH, have direct implications for genetic counseling, recurrence risk estimation, and screening for associated systemic comorbidities.[73] Therefore, although routine genetic testing is not recommended for all cases of isolated unilateral microtia, it is clearly indicated in bilateral, syndromic, or malformation-associated cases and should be incorporated into a multidisciplinary approach.[71,73,74]

HRCT of the temporal bone plays a key role in the evaluation and management of these patients, both in cases of atresia/stenosis and isolated middle ear malformations. In congenital atresia, HRCT-based scoring systems (eg, Jahrsdoerfer score) assist in selecting candidates with a higher likelihood of favorable hearing outcomes after canalplasty, ossicular chain reconstruction, and tympanic membrane reconstruction, with higher scores correlating with better prognosis.[75] A score of 5 or less disqualifies the patient for surgery, as the risks of surgery outweigh the potential benefits.[76] In isolated middle ear malformations (eg, ossicular chain anomalies, stapes footplate fixation, oval window abnormalities), HRCT can identify many structural abnormalities; however, certain fixations may not be evident on imaging and are only diagnosed during exploratory tympanotomy in selected cases.[[77], [78], [79]]

Treatment is individualized and typically combines hearing rehabilitation and **reconstructive strategies**, with decisions based on laterality, degree of hearing loss, anatomy, age, and family goals. In microtia-atresia, multiple options are available, including bone conduction PSAPs, BAHSs, and, in favorable anatomies, atresiaplasty or canalplasty, with careful coordination of timing relative to ear reconstruction.[80] For congenital middle ear malformations, the surgical approach depends on the specific anomaly (e.g., stapes fixation vs. ossicular discontinuity), often guided by intraoperative classifications (Teunissen & Cremers) and risk assessment involving the facial nerve and inner ear, with nonsurgical alternatives favored when surgical risk is high or expected hearing benefit is limited.[78,79]

Surgical correction of these malformations represents one of the most challenging procedures in otolaryngology. The goals are restoration of hearing and creation of a patent EAC.[81] However, achieving these goals is often hindered by postoperative complications, such as EAC restenosis (revision surgery may be required in 25% to 36% of cases). Conductive hearing loss remains the most common cause of hearing impairment in this population, affecting up to 33% of patients. Other potential sequelae include peripheral facial paralysis, graft lateralization, and chronic infection.[82,83]

Hearing gains vary depending on the severity of middle ear malformations and the presence of concomitant inner ear anomalies. Surgical correction is generally reserved for patients with mild malformations associated with a favorable temporal bone anatomy.[76] The use of hearing aids is feasible only in patients who underwent surgery and did not develop EAC stenosis, thus not being a good option. For other patients, there are alternative solutions for hearing rehabilitation, such as implantable systems.[84]

BAHSs are well-established options for patients with middle ear malformations, regardless of ossicular chain integrity or mobility, associated with low complication rates. Sound energy is transmitted via skull vibration directly to the cochlea, bypassing the middle ear.

The choice of implantable BAHS technology depends on the experience of the medical team. Percutaneous systems offer advantages including shorter operative time (even in malformed skulls), higher amplification capacity, fewer intraoperative complications, and smaller Magnetic Resonance Imaging (MRI) artifact in scanners up to 3.0 Tesla. However, aesthetic concerns related to the exposed abutment and the risk of skin complications and osseointegration failure, especially in young children, should be considered.

Active transcutaneous systems preserve skin integrity and are generally better accepted cosmetically, providing good hearing gain, although typically lower than that achieved with percutaneous systems. To indicate BAHSs, bone conduction thresholds must be assessed, as they are usually preserved in patients with microtia. Disadvantages include lower amplification capacity, longer operative time, larger MRI artifact, and restriction to MRI scanners up to 1.5 Tesla.

Microtia and EAC atresia exert a substantial functional impact due to conductive hearing loss and psychosocial consequences related to ear deformity, underscoring the importance of both hearing rehabilitation and ear reconstruction to restore hearing, appearance, and quality of life.[85,86] The main ear reconstruction techniques involve autologous costal cartilage or Porous Polyethylene (PPE) implants (Medpor®).[85,87] Long-term aesthetic outcomes are generally satisfactory in specialized centers, with high rates of ear symmetry, natural contour, and subjective acceptance, regardless of the material used.[87] Quality of life after reconstruction is typically high, with improvements in self-esteem and social integration. Major complications are uncommon with appropriate planning.

Hearing gains with BAHSs are robust, with hearing thresholds often improving by 30‒45 dB and speech recognition exceeding 80% in sound field testing, accompanied by high subjective satisfaction reported by patients and families.[[88], [89], [90]] Ongoing debate surrounds the optimal timing of hearing rehabilitation relative to ear reconstruction, whether they should be performed simultaneously or in staged procedures. Surgical timing depends on anatomic factors (bone thickness, mastoid development), age, reconstructive technique, family preferences, and socioeconomic factors.[85]

Comparative analyses suggest that simultaneous ear reconstruction and BAHS implantation may be advantageous by reducing total operative time, accelerating recovery, enabling earlier device activation, and not significantly increasing complication rates.[85,[88], [89], [90]] Recent studies demonstrate shorter hospital stays and earlier device activation with simultaneous protocols, without compromising wound healing or implant stability. Multidisciplinary planning is essential to avoid incision overlap, ensure flap vascularity, and position the hearing implant at a safe distance from the reconstruction site.[85,91]

Staged protocols also yield excellent aesthetic and functional outcomes and may be preferred in cases of complex anatomy, prior scarring, or the need for tissue expansion.[85] Individualized planning by a multidisciplinary team is critical to optimize outcomes and minimize risks.[91] Although technical challenges include implant exposure, local infection, and the need for revision surgery, major complication rates remain low with both approaches.

Despite growing evidence, gaps persist regarding robust long-term comparative studies between simultaneous and staged approaches, particularly in pediatric and syndromic subgroups, as well as their impact on long-term quality of life and late complications. Decision-making should therefore be individualized, based on multidisciplinary assessment, patient-specific anatomy, and expectations, with an emerging preference for simultaneous approaches when technically feasible and safe.

### Recommendations

XXVII. In unilateral malformations without immediate clinical indication, imaging studies (CT/MRI) may be deferred and scheduled according to treatment planning. *(Weak recommendation – Low-quality evidence)*

XXVIII. Children aged < 5-years with microtia/atresia should preferentially use nonsurgical hearing devices; from age 5 onward, implantable BAHSs may be considered based on anatomy and clinical context. *(Strong recommendation – Moderate-quality evidence)*

XXIX. Patients with external/middle ear malformations should undergo comprehensive clinical evaluation to screen for systemic and syndromic abnormalities. *(Strong recommendation – Low-quality evidence)*

XXX. Genetic evaluation should be performed in bilateral malformations and in syndromic/suspected cases. In isolated unilateral malformations, genetic evaluation may be selectively considered. *(Strong recommendation – Moderate-quality evidence)*

XXXI. Selection of implantable hearing technology should be based on shared decision-making with the patient/family, including discussion of benefits, limitations (including MRI), risks, self-care, and aesthetic preferences. *(Strong recommendation – Low-quality evidence)*

XXXII. Simultaneous ear reconstruction and BAHS implantation may be considered when technically feasible and safe. Staged approaches are also acceptable and may be preferred in cases of complex anatomy. *(Weak recommendation – Low-quality evidence)*

### Single-sided deafness

In adults, the CROS system is commonly offered as an initial option because it allows immediate use without the need for surgery. Although its indication in children is limited, it has proven to be a viable option in adults. Contralateral routing of sound was initially achieved by connecting a hearing aid microphone on the deaf side to a device on the normal-hearing ear.[92] Similar outcomes can now be obtained via wireless communication between 2 behind-the-ear devices.[93] Evidence suggests that CROS devices are effective in reducing the negative effects of the head shadow and improving sound awareness and SNR when sounds are directed toward the affected ear.[92,94]

BAHSs are more effective than CROS hearing aids for sound discrimination in both quiet and noise.[95] Patients may experience improvements in speech discrimination in noise, with SNR gains ranging from -3.8 to -4.8 dB, depending on sound source location.[46] However, perceived benefit and subjective hearing gain may decrease over time. Reported discontinuation rates reach 14% over 50-months, corresponding to approximately 3% per year.[96]

BAHSs can reduce the head shadow effect and improve sound awareness on the affected side.[97,98] However, they cannot restore binaural hearing or improve sound localization ability.[[97], [98], [99]] Discomfort with the device, concerns regarding sound quality, and subjective hearing difficulties are among the main reasons for rejection of both CROS and BAHS devices.[100,101] Reluctance to use sound-routing devices is also associated with changes in self-perception, aesthetic concerns, and negative social stereotypes. Despite counseling (habits, work/social environment, expectations) and extensive preoperative trials with non-implantable BAHSs, they are less effective than osseointegrated systems because signal transmission is attenuated by skin and soft tissues, especially at high frequencies, and their usable daily wear time may be limited due to discomfort from the force required to secure the device in place.[50]

Sound localization abilities depend largely on binaural cues,[102] and sound-routing devices do not restore true binaural hearing. Although it has been speculated that these devices may provide cues that allow the listener to distinguish sounds on the left from sounds on the right (lateralization) by spectrally distorting transmitted sounds, there is no evidence that devices that reroute sounds to the normal-hearing ear provide reliable cues to support spatial hearing.[103]

Surgically implantable BAHSs with superior sound quality are generally available for children aged > 5-years.[104] CROS systems present an additional limitation related to potential occlusion of the normal-hearing ear.[104] In very young children or those with narrow EACs, CROS use may be particularly challenging. Auditory deprivation associated with SSD can induce irreversible changes in the auditory cortex,[105] which neither BAHS nor CROS devices prevent, as they do not provide auditory input to the affected ear.[106]

Mertens et al.[107] evaluated 17 patients with SSD in a prospective randomized crossover study comparing an adhesive hearing system with a CROS device, each used for 2-weeks, divided into 2 groups. The results showed that 70% of participants rated the adhesive system as partially useful or better, with satisfaction levels comparable to those reported with CROS use. While sound localization improved with the adhesive system, no significant improvement was observed in speech perception in noise. Another study involving 9 patients with SSD evaluated a device transmitting vibration through the palate and showed improvements in speech recognition and overall quality of life in both quiet and noisy environments.[108]

Regarding quality of life, a systematic review and meta-analysis found that BAHS use in adults with SSD was associated with significant improvements in hearing-related quality of life, whereas no significant differences were observed in generic quality-of-life measures assessed by the Health Utilities Index Mark-3 (HUI-3).[109]

Evidence suggests that sound-routing devices may improve speech perception in noise under specific listening conditions. Statistically significant benefits have been demonstrated primarily when the SNR is more favorable on the deaf side than on the normal-hearing side. In this scenario, sound-routing devices increase the SNR in the normal ear by overcoming the head shadow effect.[110] Reported BAHS-related improvements in SNR range from 0.4 dB[110] to 4.4 dB,[111] highlighting substantial heterogeneity and leaving uncertainty regarding the clinical relevance of these gains.

### Recommendations

XXXIII. In adults with SSD, the CROS system may be offered as an initial option because it is immediate and nonsurgical, with realistic counseling regarding its limitations. *(Weak recommendation – Moderate-quality evidence)*

XXXIV. BAHS and CROS devices may improve speech perception in noise in specific scenarios (when the SNR is more favorable on the deaf side); however, they do not restore binaural hearing or sound localization. Device selection should be patient-centered. *(Weak recommendation – Moderate-quality evidence)*

XXXV. In children with SSD, the indication for sound-routing devices should be cautious, recognizing limitations in auditory development and the need for long-term monitoring. *(Strong recommendation – Low-quality evidence)*

XXXVI. In pediatric SSD, imaging (preferably high-resolution MRI) should be performed due to the elevated risk of cochlear nerve deficiency and other malformations. *(Strong recommendation – Moderate-quality evidence)*

XXXVII. The contralateral ear in patients with SSD should be followed longitudinally because of the risk of delayed-onset hearing loss. *(Strong recommendation – Low-quality evidence)*

### Bahs in patients with tinnitus

Among candidates for BAHS, whether due to conductive/mixed hearing loss or SSD, tinnitus is a frequent complaint. By restoring or optimizing auditory input, and potentially providing masking or sound enrichment effects, bone-conduction stimulation may reduce tinnitus perception or distress in a subset of patients. Bone conduction approaches have therefore been explored as a potential strategy for tinnitus relief, with studies suggesting possible benefit.[112]

Most available evidence derives from clinical series and patient-reported outcome studies involving individuals with conductive or mixed hearing loss rehabilitated with BAHSs. Tinnitus and quality of life are typically secondary outcomes. BAHS rehabilitation has been associated with improvements in quality of life and, in selected subgroups, reductions in tinnitus severity; however, results are heterogeneous (differences in tinnitus etiology, assessment instruments, and the frequent absence of control groups).[113,114] Systematic reviews focusing on percutaneous systems consistently demonstrate audiological and quality-of-life benefits but do not support tinnitus as an isolated primary indication.

In patients with SSD, among whom tinnitus may be particularly disabling, higher-level evidence is available. A randomized controlled trial comparing cochlear implant, BAHS, CROS, and no treatment showed a significant reduction in tinnitus impact scores in both the cochlear implant and BAHS groups (with a greater magnitude of effect in the cochlear implant group), with follow-up extending to 24-months.[115] Systematic reviews and meta-analyses in SSD populations discuss tinnitus suppression as a relevant outcome and suggest that hearing rehabilitation interventions may alleviate symptoms, although results vary according to the modality and study design.[109,116]

BAHSs are not considered a first-line treatment for primary tinnitus.[117] While tinnitus may improve when BAHSs are indicated for hearing rehabilitation, these devices should be offered only when a clear audiological indication exists and when patients have realistic expectations regarding tinnitus outcomes.[115,117]

### Recommendations

XXXVIII. BAHS should not be indicated as a primary treatment for tinnitus in patients with SSD and profound unilateral hearing loss. *(Strong recommendation – Low-quality evidence)*

XXXIX. In patients with conductive or mixed hearing loss who meet audiological criteria for BAHS, the presence of tinnitus does not contraindicate implantation and may provide improvements; realistic counseling regarding expectations is essential. *(Strong recommendation – Low-quality evidence)*

### Cochlear nerve hypoplasia

Hypoplasia or aplasia of the cochlear nerve is frequently associated with inner ear malformations, particularly common cavity deformity and incomplete partition type I, and may also occur in cases of incomplete partition type II. In addition, hypoplasia of the Internal Auditory Canal (IAC) represents an important anatomic marker associated with Cochlear Nerve Deficiency (CND).[118] Reported prevalence of CND varies widely in the literature, ranging from 9% to 50%.[[119], [120], [121], [122]] In pediatric populations, CND is significantly more prevalent in unilateral hearing loss, with reported rates of 39% to 50%, compared with approximately 5% in bilateral hearing loss.[123,124]

Diagnostic evaluation relies primarily on MRI, particularly parasagittal views of the IAC, which allow direct visualization of the cochlear nerve relative to the other vestibular-facial nerve bundles.[125,126] HRCT of the temporal bone plays a complementary role by identifying IAC stenosis and associated inner ear malformations.[125] Electrophysiologic testing, such as ABR, frequently shows absent responses but cannot reliably distinguish between nerve hypoplasia and aplasia, reinforcing the key role of MRI for definitive diagnosis.[20] HRCT of the temporal bone or MRI of the brain are most commonly performed. Malformations are identified on HRCT and/or MRI in approximately 25% to 67% of patients with unilateral hearing loss,[121,127] with reported rates ranging from 3% to 50% in studies limited to SSD populations.[127]

CND exists along a spectrum from complete aplasia to hypoplasia. The cochlear nerve is best assessed using high-resolution T2-weighted MRI,[128] which has increased CND diagnostic rates. CND may be present on MRI even when the cochlear aperture or IAC appear normal.[120,129] Although no universal diagnostic cutoff has been established, recent studies have demonstrated a strong association between CND and Cochlear Aperture Stenosis (CAS), defined as an aperture width ranging from < 1.4 to < 1.7 mm, suggesting CAS as a potential predictor of CND.[119,130]

The relationship between hearing thresholds and CND remains poorly characterized. Most patients exhibit profound hearing loss or absent threshold responses regardless of the use of hearing aids.[118,126,131] However, ears with imaging evidence of CND may demonstrate mild to moderate hearing loss, indicating that hearing thresholds alone are unreliable predictors of functional cochlear nerve integrity.[126.132]

Hearing rehabilitation in CND depends on laterality and the **type of neural malformation**. In unilateral CND, particularly in cases of cochlear nerve hypoplasia, hearing aids may provide limited benefit, mainly for sound detection and support for early auditory development, although performance in noise remains compromised.[[119], [120], [121], [122]] In bilateral cochlear nerve hypoplasia, cochlear implant may be considered selectively, but outcomes are highly variable and generally inferior to those in children with a normal cochlear nerve; the development of functional oral language remains unpredictable.[122,133]

In cases of bilateral cochlear nerve aplasia, cochlear implant may be ineffective, and Auditory Brainstem Implant (ABI) represents an alternative rehabilitation strategy.[134] Although outcomes with ABI in children without neurofibromatosis type 2 have improved over the past decades, benefits are largely limited to environmental sound awareness and support for multimodal communication.[118,126,131] Given the frequent association of bilateral CND with genetic syndromes and neurodevelopmental disorders, genetic evaluation is strongly recommended to inform family counseling, prognosis, and multidisciplinary treatment planning.[126,132]

In unilateral hearing loss, BAHS may be considered when conventional hearing aids provide no benefit or when the cochlear nerve is nonfunctional. BAHS should be compared with other options, such as conventional hearing aids, CROS systems, and cochlear implant. In SSD, BAHS and CROS represent alternatives when cochlear implant is contraindicated or when there is no functional neural response.[20,98,135,136]

Comparative studies indicate that both CROS and BAHS can improve speech perception in noise, particularly when the sound source is directed toward the affected side.[135,136] No significant differences have been demonstrated between the two systems in objective speech-in-noise outcomes, although BAHS may yield higher subjective satisfaction in some patients. Both approaches fail to restore binaural hearing or sound localization.[98,135,137] Device selection should consider patient age, anatomy, maturity, family expectations, and surgical risk.[98,135,137]

In children with cochlear nerve hypoplasia and profound unilateral hearing loss, CROS or BAHS systems may be considered when conventional hearing aids are ineffective, cochlear implant is contraindicated, or anatomic malformations are present.

Decision-making should be guided by a multidisciplinary assessment incorporating anatomy, maturity, family expectations, and surgical risk. BAHS selection should consider: 1) Degree and type of hearing loss (conductive, mixed, or profound unilateral/bilateral sensorineural); 2) Presence/absence of the cochlear nerve (confirmed by imaging and functional tests); 3) Hearing results (thresholds, speech recognition in quiet and noise); and 4) Temporal bone anatomy and surgical feasibility.

Family counseling is essential, emphasizing realistic discussion of benefits, limitations, and risks. Decisions should be individualized and involve a multidisciplinary team, considering anatomic, functional, and subjective factors. Currently, no formal guidelines specifically address BAHS use in cochlear nerve hypoplasia; recommendations are based on expert consensus and available evidence.[20,98] Important gaps remain in the literature, particularly the lack of robust long-term studies involving young children with cochlear nerve hypoplasia and direct comparisons between CROS and BAHS regarding functional, cognitive, and quality-of-life outcomes.

### Recommendations

XL. For the diagnosis of CND, MRI (high-resolution T2-weighted sequences, including parasagittal views of the IAC) should be performed; HRCT should be used as a complementary modality to assess the IAC, cochlear aperture, and associated malformations. *(Strong recommendation – Moderate-quality evidence)*

XLI. In SSD with CND or a nonfunctional nerve, or when cochlear implant is not indicated, BAHS or CROS may be considered as alternatives, with individualized decision-making and realistic counseling. *(Weak recommendation – Low-quality evidence)*

### Chronic otitis media

The overall incidence of Chronic Otitis Media (COM) is estimated at 4.76%.[138] It is characterized by symptoms such as aural fullness, tinnitus, pruritus, otalgia, recurrent otorrhea, and, most importantly, hearing loss. Approximately 31 million new cases are diagnosed annually, with the majority occurring in low- and middle-income countries: nearly one-fifth affect children younger than 5-years. COM may lead to progressive middle ear damage and typically causes conductive hearing loss due to tympanic membrane perforation, reduced tympanic membrane mobility, and/or ossicular chain discontinuity. However, disease-related complications have also been associated with sensorineural hearing loss.[139]

Hearing rehabilitation is more difficult to achieve in patients with cholesteatoma than in those without the disease. This is likely related to poor middle ear aeration, the high frequency of ossicular chain damage in cholesteatoma, and the challenges associated with restoring ossicular continuity.[140] However, these data should be interpreted cautiously, given the heterogeneity of underlying disease across subgroups.

Hearing rehabilitation in patients with COM should be planned in parallel with disease control (particularly in the presence of cholesteatoma) and with consideration of the degree of preservation of middle ear structures. Management strategies should integrate ear status (dry vs. active otorrhea), type and degree of hearing loss (conductive, mixed, or sensorineural), postoperative anatomy (cavity configuration, ossicular chain status, ventilation), risk of recurrence, and the need for long-term follow-up.[141]

Two key issues complicate hearing rehabilitation in patients with COM: 1) Surgical intervention does not always achieve complete air-bone gap closure, even in the short term, and 2) A sensorineural component may be present or may develop over time as a consequence of chronic inflammation. A systematic review of tympanoplasty outcomes in COM showed that, at follow-up ≥ 12-months, approximately 70% of patients achieved an air-bone gap < 20 dB, with an overall complication rate of 14% (varying by technique, presence of cholesteatoma, and need for ossiculoplasty).[142] Even in “uncomplicated” COM, classical evidence indicates deterioration of bone-conduction thresholds (clinically relevant in a subset of patients), correlating with disease type and duration, which directly influences the choice between air-conduction rehabilitation and bone-conduction alternatives.[143]

Before considering electronic hearing devices for rehabilitation in COM, conventional middle ear surgical interventions should be evaluated and prioritized whenever feasible. Ossiculoplasty aims to restore mechanical sound transmission and can significantly reduce the air-bone gap, with reported success rates (air-bone gap ≤20 dB) of 60% to 80% in selected clinical series, particularly in dry ears with favorable anatomy.[144,145] The use of a Partial Ossicular Replacement Prosthesis (PORP) or a Total Ossicular Replacement Prosthesis (TORP), manufactured from biocompatible materials such as titanium, has yielded consistent functional hearing outcomes in COM.[[146], [147], [148]]

Key prognostic factors include the integrity of the stapes and status of the middle ear mucosa, commonly assessed using indices such as the Middle Ear Risk Index (MERI) and the Ossiculoplasty Outcome Parameter Staging (OOPS).[149] MERI and OOPS provide complementary prognostic stratification in middle ear surgery but differ fundamentally in scope. MERI integrates clinical and intraoperative factors reflecting the degree of inflammatory activity and anatomic destruction of the middle ear, including persistent otorrhea, cholesteatoma, granulation tissue, ossicular chain integrity, prior surgery, and, in later versions, smoking. By assigning greater weight to markers of active disease, MERI correlates consistently with poorer anatomic and hearing outcomes after tympanoplasty and is particularly useful for characterizing case complexity and surgical environment.

OOPS was developed to more specifically predict functional outcomes after ossiculoplasty. It emphasizes parameters directly related to ossicular reconstruction and sound transmission, such as middle ear mucosal condition, presence of the malleus, active drainage, type of mastoidectomy (canal wall up vs. canal wall down), and revision surgery. With a simpler and more targeted scoring system, OOPS demonstrates stronger correlation with air-bone gap closure and is more sensitive in discriminating hearing prognosis across different ossiculoplasty techniques and prostheses. Thus, while MERI offers a comprehensive assessment of middle ear disease severity, OOPS complements it by providing a more refined estimate of expected hearing gain, particularly when functional outcomes are the primary endpoint.

OOPS incorporates the type of mastoidectomy as a unique variable and demonstrates a stronger correlation with post-ossiculoplasty hearing outcomes in patients with COM undergoing mastoidectomy. In contrast, MERI provides a more comprehensive assessment of surgical risk but is less specific in predicting postoperative hearing outcomes in these cases.

Late complications after ossiculoplasty occur in approximately 10% of cases and include prosthesis extrusion (3%‒8%), displacement, tympanic graft failure, and cholesteatoma recurrence. Revision surgery is required in 8% to 16% of cases, with higher rates among patients with Eustachian tube dysfunction or sclerotic mastoids. Quality of life improves when stable hearing gain is achieved but may be limited by disease recurrence or functional failure.[149,150]

Conventional PSAPs are effective in patients with a dry ear and without extensive perforation but frequently cause irritation or perpetuate otorrhea in unstable cavities, limiting use and negatively affecting quality of life. In the presence of persistent perforation or otorrhea, their use is contraindicated, and canal-related complication rates are high. The use of PSAPs depends on middle ear anatomic stability. Rehabilitation strategies should consider factors such as otorrhea frequency, tolerance to the use of ear molds or domes, need for frequent local care, occupational or recreational water exposure, and realistic expectations of hearing benefits.[141]

BAHSs and BCIs provide significant and sustained improvements in hearing and speech discrimination, with benefits maintained in the long term. Late complication rates are low, and the need for revision surgery is rare. Patients report improved quality of life, consistent device use, and reduced impact of otorrhea or mastoid cavity instability. These systems represent the preferred option when conventional PSAPs are contraindicated after radical mastoidectomy.[151,152]

BAHSs yield superior long-term hearing and quality-of-life outcomes with lower complication and revision rates, particularly in patients unable to use conventional PSAPs after mastoidectomy.[[151], [152], [153]]

Reports describe mastoidectomy combined with osseointegrated device implantation in single-stage or two-stage procedures. Ardıç et al.[154] demonstrated favorable outcomes in patients with COM who have undergone surgical treatment, without increased postoperative infection rates. In cases of complex temporal bone anatomy, such as large cavities or anteriorly located sigmoid sinus, alternative approaches (middle fossa or retrosigmoid) have been shown to be safe. These findings suggest that hearing rehabilitation may be combined with surgical treatment of middle ear disease in selected cases, provided infection is controlled. Despite advances, important gaps remain ‒ particularly the lack of independent randomized trials directly comparing these technologies in COM ‒ limiting the development of universal protocols.

## Cholesteatoma

In patients undergoing subtotal petrosectomy for cholesteatomatous COM, imaging follow-up with MRI is mandatory. Non-echo-planar diffusion-weighted imaging (non-EPI DWI) is currently considered the modality of choice for detecting residual or recurrent disease after middle ear surgery.[141] Paradoxically, this diagnostic modality is also highly susceptible to degradation from metallic and magnetic artifacts. Experimental and clinical studies have shown that transcutaneous bone-conduction implants, particularly those incorporating magnets or active components, may generate extensive artifacts on MRI, rendering ipsilateral non-EPI DWI sequences nondiagnostic. In comparative studies at 1.5 Tesla, the Osia 2 system (magnet-free) produced the least artifact compared with BCI systems.[155]

Although, to date, no clinical studies have directly evaluated the impact of such artifacts specifically after subtotal petrosectomy combined with hearing rehabilitation, surgical series and technical reviews in cochlear implant emphasize that imaging surveillance is integral to patient management, and the rehabilitation strategy should be compatible with planned imaging follow-up.[156,157]

When the need for serial MRI is anticipated and the surveillance target is ipsilateral, solutions that minimize artifact generation or, alternatively, non-implantable options ‒ whether temporary or definitive ‒ may be preferred, particularly when cutaneous or osseous conditions are unfavorable. This approach aims to preserve diagnostic accuracy of imaging surveillance while optimizing long-term management.

### Recommendations

XLII. In COM, the hearing rehabilitation strategy should be planned in parallel with disease control, considering ear status, postoperative anatomy, recurrence risk, and follow-up requirements. *(Strong recommendation – Moderate-quality evidence)*

XLIII. Middle ear reconstructive interventions (tympanoplasty/ossiculoplasty) should be evaluated and prioritized when feasible before electronic hearing devices. *(Strong recommendation – Moderate-quality evidence)*

XLIV. In patients with unstable radical cavity (persistent otorrhea) and contraindication or intolerance to air-conduction PSAPs, BAHSs should be considered the preferred rehabilitation option. *(Strong recommendation – Moderate-quality evidence)*

XLV. In patients with a predictable need for serial MRI (eg, cholesteatoma surveillance with non-EPI DWI, subtotal petrosectomy), device selection should consider the impact of the artifact on the ipsilateral side. Solutions associated with less artifact (percutaneous devices) or non-implantable solutions may be preferred when diagnostic imaging is critical. *(Strong recommendation – Low-quality evidence)*

### Otosclerosis

The surgical procedure indicated for hearing rehabilitation in patients with otosclerosis is stapedotomy, which is considered safe when performed by an experienced surgeon.[158,159] There are few indications for the use of active middle ear implants or BAHSs in patients with otosclerosis. Disease progression may worsen hearing thresholds to levels that no longer meet candidacy criteria for these devices, and most patients adapt satisfactorily to conventional hearing aids. Limited indications include: 1) Unfavorable surgical anatomy (persistent stapedial artery, obliteration of the oval window by a dehiscent facial nerve); 2) Otosclerotic involvement in an only-hearing ear; and 3) Concern for occupational functional loss due to complications or stapes surgery.

### Osteogenesis imperfecta

Hearing loss is a significant manifestation of Osteogenesis Imperfecta (OI), particularly OI type I, the most prevalent form, associated with quantitative mutations in the *COL1A1* gene. Clinical features include recurrent fractures, skeletal deformities, blue sclera, dentinogenesis imperfecta, and, in many cases, progressive hearing loss. Up to 50%–60% of adults with OI type I develop progressive hearing loss, which may be conductive, sensorineural, or mixed. The prevalence of hearing loss in OI is estimated to be as high as 70%, varying by age and genetic subtype.[160] Ossicular chain involvement and otic capsule abnormalities contribute to the conductive component, whereas cochlear degeneration associated with type I collagen deficiency accounts for the sensorineural component.[161]

Traditional surgical approaches, such as stapedotomy, yield unpredictable results in patients with OI due to bone fragility and the risk of ossicular fracture. A meta-analysis reported successful air-bone gap closure (≤ 10 dB) in only approximately 59% of cases of stapes surgery in OI, a rate lower than that observed in nonsyndromic otosclerosis.[162] Implantable hearing devices, associated with lower surgical morbidity and risk, particularly in patients with conductive or mixed hearing loss who do not benefit from conventional hearing aids, may be considered in selected cases. In patients with OI, although feasible, percutaneous and passive transcutaneous systems should be used with caution because of the unpredictability of osseointegration and potential device failure.[163,164]

Active transcutaneous systems overcome these limitations, as the transducer is implanted under the temporal bone and transmits sound via internal vibration, without reliance on osseointegration for acoustic conduction.[8] Comparative studies suggest that these systems provide hearing performance comparable to percutaneous devices, with lower rates of skin complications and improved long-term stability.[165] In patients with OI, particularly moderate to severe forms, active transcutaneous systems may therefore represent a more suitable option due to their reduced dependence on local bone quality.

### Recommendations

XLVI. In otosclerosis, stapedotomy should remain the standard strategy when indicated and performed by an experienced surgeon. BAHSs/active implants should be reserved for exceptional situations (unfavorable anatomy, only-hearing ear, occupational risk). *(Strong recommendation – Moderate-quality evidence)*

XLVII. In OI, percutaneous and passive transcutaneous systems should be indicated with caution because of unpredictable osseointegration. Active transcutaneous systems may be preferred when BAHS is required. *(Strong recommendation – Low-quality evidence)*

### Temporal bone tumors

In patients with cancer undergoing skull base and/or middle ear resections, particularly those with previously irradiated bone and skin, the selection of a hearing rehabilitation strategy requires a balanced assessment across 3 fundamental axes: 1) The need for serial MRI follow-up, including surveillance of the IAC, Cerebellopontine Angle (CPA), and tumor bed; 2) The risk of skin complications; and 3) The feasibility of osseointegration in biologically compromised tissues.

Percutaneous systems with an abutment provide efficient sound transmission and, from an imaging standpoint, are generally more MRI-friendly because they do not use magnets, resulting in less device-related artifact. However, they depend directly on adequate bone and skin quality for sustained healing ‒ an especially critical consideration in irradiated patients.[166]

Radiotherapy to the temporal bone induces microvasculopathy, fibrosis, and skin fragility, increasing the risk of wound dehiscence, necrosis, infection, and osteoradionecrosis, thereby making the timing of implantation a key decision point. Placement of osseointegrated devices before radiotherapy, ideally at the time of cancer surgery, has been associated with lower rates and severity of complications compared with delayed implantation.[167,168] Irradiated patients with osseointegrated hearing devices exhibit a significantly increased long-term risk of complications involving both soft tissues and the implant, with a cumulative effect over post-radiotherapy follow-up and a possible association with higher radiation doses.[169]

Passive transcutaneous systems also require osseointegration and, in addition, exert continuous pressure on irradiated skin via magnetic coupling. Case reports and case series have documented skin complications related to magnet adjustment, including necrosis in Baha Attract systems, while comparative studies between Sophono and Baha Attract have highlighted the skin as a critical element requiring careful fine-tuning in these devices.[59,170,171] In irradiated patients, passive transcutaneous systems may represent a particularly unfavorable option by simultaneously requiring osseointegration and reduced skin tolerance to sustained pressure.

The need for MRI surveillance constitutes a second crucial decision axis. Active transcutaneous systems, such as Bonebridge and Osia, are generally classified as MRI-conditional under specific conditions; however, the central issue extends beyond examination safety to the impact of imaging artifacts on the anatomic region of interest. Depending on device, implant position, and imaging sequence, artifacts may preclude adequate evaluation of the ipsilateral temporal bone and IAC/CPA. For Bonebridge, studies have demonstrated the presence of extensive artifacts, although artifact-reduction techniques and careful implant site planning may improve IAC visualization, an important consideration when tumor surveillance is a priority.[172,173]

For the Osia system, the presence of a magnet contributes substantially to imaging artifacts. Temporary magnet removal may be decisive for obtaining diagnostically adequate diffusion-weighted images of the ipsilateral temporal bone, and there is a case report describing bilateral magnet displacement during MRI in a patient with an Osia implant.[174] Post-marketing surveillance analyses have also identified reports of adverse events associated with active bone-conduction implants in multiple clinical settings, including MRI examinations. However, the nature of these data does not allow determination of incidence or a direct causal relationship.[175]

These data support an individualized approach to bone-conduction hearing rehabilitation in irradiated patients and those with bone disease, taking into account imaging objectives, skin condition, timing relative to radiotherapy, and tolerance for local complication risk.

### Recommendations

XLVIII. In irradiated patients or those with temporal bone tumors, the choice of bone-conduction hearing rehabilitation should be individualized, considering the need for diagnostic MRI (artifact), risk of skin complications/osteoradionecrosis, and feasibility of osseointegration. When a percutaneous device is indicated, placement of the abutment before or at the time of cancer surgery may be considered. *(Weak recommendation – Low-quality evidence)*

### Complications

The main disadvantage of percutaneous BAHSs is the higher risk of skin-related problems, extrusion, and need for revision surgery. Complication rates vary widely and are influenced by surgical technique, surgeon experience, device design, patient age, and predisposing factors for infection or impaired wound healing. Meticulous care of the skin surrounding the abutment is essential. In 1988, Holgers et al.[54] published a study following 60 patients with percutaneous BAHSs and proposed a grading system to classify skin complications and guide treatment; this system remains widely used today.

Mohamad et al.[176] published a systematic review of 30 studies involving percutaneous BAHS users and showed skin complication rates of 9.4% to 84%. A meta-analysis by Kiringoda and Lustig,[177] including 2310 implants, found Holgers grade 2 or higher skin reactions ranging from 2.4% to 38.1%, and revision surgery rates ranging from 1.7% to 34.5% in adults and 0% to 44.4% in pediatric patients.

Skin reactions typically occur early, within the first 12-months postoperatively, but may appear up to 46-months (3.3-years) after surgery.[31,35] A Holgers grade 2 or higher warrants treatment.[178] Reactions graded as Holgers 2 or higher occur in approximately 5% to 15% of patients. On average, 0.4% of patients develop Holgers grade 4 reactions (usually later complications) requiring systemic antibiotic therapy. Reported complication rates vary according to the medical team’s experience with the procedure and device manufacturer.[51,177]

Abutment loss was reported in 8.3% of patients in a large cohort of more than 1000 implanted percutaneous bone-conduction devices.[35] A meta-analysis reported rates ranging from 2% to 17%.[177] Higher rates are observed in pediatric patients and in individuals aged > 60-years, likely due to reduced bone thickness and density, diminished bone vascularity, nutritional deficiencies, smoking, renal failure, prior radiotherapy, and increased risk of postoperative trauma, in addition to inadequate surgical technique (lack of irrigation during drilling, excessive heat generation, or inappropriate drilling speed).[4]

Children exhibit significantly higher rates of implant loss (up to 18% in children vs. 2.5% in adults), osseointegration failure, and revision surgery.[177,179] Skin complications, such as infections, adverse skin reactions (Holgers grade ≥ 2), and soft tissue hyperplasia, are common, with up to 44% of children requiring revision procedures, especially those younger than 5-years or with genetic syndromes.[180] Syndromic children are at an even greater risk of implant extrusion and severe skin reactions. Furthermore, postoperative follow-up in children should be more rigorous and frequent due to greater instability of the implant and susceptibility to complications, requiring meticulous care.[180] Although novel surgical techniques and larger-diameter implants may partially reduce these complications, between-study variance remains high and the risk in children remains higher than in adults.

In children, transcutaneous systems demonstrate significantly lower rates of skin complications, implant loss, and revision surgery compared with percutaneous systems (skin complications: 6.3% vs. 30%; implant loss: 0% vs. 5.3%; revisions: 3% vs. 6.2%).[10,36,181] Although percutaneous devices may offer slightly superior hearing gain (mean difference of 4‒6 dB in pure-tone and speech thresholds), the increased risk of infection, extrusion, severe skin reactions, and greater postoperative care burden favors transcutaneous systems, especially in syndromic children, who are at an even greater risk of serious complications.[10,62,181,182] Recent advances in transcutaneous devices, such as thinner transducers and improved anatomic conformity, have expanded their safety and efficacy in young children, including those younger than 5 years, with high satisfaction and low adverse event rates.

Passive and active transcutaneous systems provide comparable hearing benefits, with mean functional gains of 31‒41 dB and substantial improvements in speech perception in noise, while exhibiting significantly lower rates of skin complications and revision surgery.[183,184] Recent studies suggest that the Osia system offers superior high-frequency gain and accommodates poorer bone-conduction thresholds in regular users.[185,186] Comparative studies have not demonstrated statistically significant differences in overall hearing outcomes or quality-of-life improvements between Bonebridge and Osia, although Osia may provide better high-frequency performance and Bonebridge slightly better speech understanding at 50% discrimination.[8,[184], [185], [186]]

### Recommendations

XLIX. In users of percutaneous BAHS, meticulous and systematic care of the skin surrounding the abutment is essential, with structured education for patients/family regarding hygiene and early signs of complications. *(Strong recommendation – Low-quality evidence)*

L. The Holgers grading system should be used to classify and manage skin reactions in percutaneous systems; a grade ≥2 should prompt active treatment. *(Strong recommendation – Moderate-quality evidence)*

LI. Patients with percutaneous BAHS should be followed more intensively during the first 12‒36 months postoperatively, the period associated with the highest incidence of skin reactions and implant failures. *(Strong recommendation – Low-quality evidence)*

LII. In children, especially those aged < 5-years and/or with syndromic conditions, percutaneous BAHS should be indicated with caution due to the higher risk of osseointegration failure, extrusion, and need for revision surgery. *(Strong recommendation – Moderate-quality evidence)*

LIII. In pediatric candidates for bone-conduction hearing rehabilitation, transcutaneous systems (passive or active) should be prioritized when feasible, given their lower rates of skin complications, implant loss, and revision surgery. *(Strong recommendation – Moderate-quality evidence)*

LIV. In patients with risk factors for impaired wound healing (extremes of age, genetic syndromes, smoking, renal failure, prior radiotherapy, skin fragility), percutaneous systems should be avoided or indicated only after careful risk-benefit assessment. *(Strong recommendation – Low-quality evidence)*

LV. Passive and active transcutaneous systems should be considered preferred alternatives to percutaneous devices when the primary goal includes reducing skin complications and the need for revision surgery, accepting potential small audiometric differences. *(Strong recommendation – Moderate-quality evidence)*

LVI. Minor hearing advantages favoring percutaneous systems (≈4‒6 dB) should not, alone, justify their selection when the risk of skin complications and revision surgery is high. *(Strong recommendation – Moderate-quality evidence)*

LVII. Among transcutaneous systems, the choice between passive and active devices should consider bone-conduction thresholds, the need for high-frequency gain, and device tolerability, as consistent differences in quality of life have not been demonstrated between Bonebridge and Osia. *(Strong recommendation – Moderate-quality evidence)*

LVIII. Osia may be preferred in patients with greater high-frequency demands or higher bone-conduction thresholds compared with other transcutaneous systems. *(Weak recommendation – Moderate-quality evidence)*

### Quality of life

Recent cost-effectiveness analyses indicate that the long-term costs of the Bonebridge and percutaneous devices converge over time, primarily due to the lower rates of skin complications and revision surgery associated with Bonebridge. A 5-year longitudinal study showed that, although Bonebridge entails higher upfront costs, total cumulative costs at 5-years were statistically comparable to those of percutaneous devices, while providing lower complication rates and similar hearing benefits and patient satisfaction.[183] A study conducted in Sweden using a Markov model demonstrated that Osia is cost-effective compared with percutaneous devices, with an incremental cost-effectiveness ratio of 108,318 SEK per Quality-Adjusted Life Year (QALY) gained, driven by fewer complications, higher utility scores, and improved cosmetic outcomes.[65]

Pediatric data further support a lower health care burden associated with transcutaneous systems, including fewer outpatient visits, skin complications, and revision surgeries compared with percutaneous systems.[185] Current-generation percutaneous implants demonstrate improved cost-effectiveness relative to earlier designs, with additional savings achievable through enhanced implant survival.[55]

Meta-analyses and cohort studies consistently report higher quality-of-life scores for transcutaneous devices compared with percutaneous systems, with significant differences observed in validated instruments such as the Glasgow Benefit Inventory and the Abbreviated Profile of Hearing Aid Benefit.[181] Both Bonebridge and Osia provide substantial hearing benefits, improved speech recognition, and high patient satisfaction.[5,187]

### Recommendations

LIX. Quality-of-life assessment should be integrated into treatment decision-making when selecting a BAHS, using validated instruments whenever possible. *(Strong recommendation – Low-quality evidence)*

LX. In long-term analyses, active transcutaneous systems should be considered cost-effective compared with percutaneous systems, particularly when considering reduced rates of complications and revision surgery. *(Strong recommendation – Moderate-quality evidence)*

LXI. In pediatric populations, the lower health care burden associated with transcutaneous systems should be considered a relevant factor in technology selection. *(Strong recommendation – Moderate-quality evidence)*

## Conclusion

All types of BAHSs provide significant and sustained benefits, and device selection should be individualized according to the patient’s audiological profile, anatomic considerations, and personal preferences.[17,181,185]

Active transcutaneous devices are more cost-effective for health care systems and demonstrate superior long-term safety and quality-of-life outcomes compared with percutaneous devices,[65,180,182] but with no statistically significant difference in quality of life when compared to each other.[185]

## ORCID ID

Rogério Hamerschmidt: 0000-0002-7722-6409

Felippe Felix: 0000-0001-5314-6546

Robinson Koji Tsuji: 0000-0001-5164-0786

Mariana Moreira de Castro Denaro: 0000-0003-4331-6438

Pauliana Lamounier: 0000-0003-3552-5264

Arthur Menino Castilho: 0000-0002-9024-8004

Vagner Antonio Rodrigues Silva: 0000-0002-7335-4489

## Conflicts of interest

The authors declare no conflicts of interest.

## Data availability statement

The authors declare that all data are available in repository.

## References

[1] Stenfelt S., Puria S., Hato N., Goode R.L. (2003). Basilar membrane and osseous spiral lamina motion in human cadavers with air and bone conduction stimuli. Hear Res..

[2] Stenfelt S., Goode R.L. (2005). Bone-conducted sound: physiological and clinical aspects. Otol Neurotol..

[3] da Silva V.A.R., Kruchewsc M.M., Lavinsky J. (2021). Progressive asymmetry in occupational noise-induced hearing loss: a large population-based cohort study with a 15-year follow-up. J Int Adv Otol..

[4] Lee J.W.Y., Bance M.L. (2019). Physiology of osseointegration. Otolaryngol Clin North Am..

[5] Zou Y., Wang Q., Liu C., Ren L., Sun X., Yang S. (2023). Precise selection of bone conduction hearing devices for congenital malformation of the middle and outer ear (CMMOE). Acta Otolaryngol..

[6] Syms M.J., Hernandez K.E. (2014). Bone conduction hearing: device auditory capability to aid in device selection. Otolaryngol Head Neck Surg..

[7] Skarzynski P.H., Ratuszniak A., Osinska K. (2019). A comparative study of a novel adhesive bone conduction device and conventional treatment options for conductive hearing loss. Otol Neurotol..

[8] Caversaccio M., Wimmer W., Hoch A., Dejaco T., Schwab B. (2025). Safety profiles of bone-conduction hearing implants revisited: A meta-analytic comparison adjusted for follow-up time. Eur Arch Otorhinolaryngol..

[9] Cooper T., McDonald B., Ho A. (2017). Passive transcutaneous bone conduction hearing implants: a systematic review. Otol Neurotol..

[10] Gutierrez J.A., Shannon C.M., Nguyen S.A., Meyer T.A., Lambert P.R. (2024). Comparison of transcutaneous and percutaneous implantable hearing devices for the management of congenital aural atresia: a systematic review and meta-analysis. Otol Neurotol..

[11] Arberas C., Ruggieri V. (2013). [Autism and epigenetics. A model of explanation for the understanding of the genesis in autism spectrum disorders]. Medicina (B Aires)..

[12] Ghoncheh M., Stenfelt S., Maas P. (2022). Output performance of the novel active transcutaneous bone conduction implant Sentio at different stimulation sites. Hear Res..

[13] Qaseem A., Snow V., Owens D.K., Shekelle P., Physicians CGCotACo (2010). The development of clinical practice guidelines and guidance statements of the American College of Physicians: summary of methods. Ann Intern Med..

[14] Schünemann H.J., Schünemann A.H., Oxman A.D. (2008). Grading quality of evidence and strength of recommendations for diagnostic tests and strategies. BMJ..

[15] Haugen B.R., Alexander E.K., Bible K.C. (2016). 2015 American thyroid association management guidelines for adult patients with thyroid nodules and differentiated thyroid cancer: the american thyroid association guidelines task force on thyroid nodules and differentiated thyroid cancer. Thyroid..

[16] Bossuyt P.M., Reitsma J.B., Bruns D.E. (2003). Towards complete and accurate reporting of studies of diagnostic accuracy: The STARD Initiative. Ann Intern Med..

[17] Portelli D., Ciodaro F., Loteta S., Alberti G., Bruno R. (2023). Audiological assessment with Matrix sentence test of percutaneous vs transcutaneous bone-anchored hearing aids: a pilot study. Eur Arch Otorhinolaryngol..

[18] Dalè M., Buono F., Simoni F. (2025). Critical issues in bone anchored hearing aids. Eur Arch Otorhinolaryngol..

[19] Snik A.F., Mylanus E.A., Proops D.W. (2005). Consensus statements on the BAHA system: where do we stand at present?. Ann Otol Rhinol Laryngol Suppl..

[20] Tsuji R.K., Hamerschmidt R., Lavinsky J., Felix F., Silva V.A.R. (2025). Brazilian Society of Otology task force - single sided deafness - recommendations based on strength of evidence. Braz J Otorhinolaryngol..

[21] Silva V.A.R., Pauna H.F., Lavinsky J. (2023). Task force Guideline of Brazilian Society of Otology ‒ hearing loss in children - Part I ‒ Evaluation. Braz J Otorhinolaryngol..

[22] Sprinzl G.M., Wolf-Magele A. (2016). The Bonebridge bone conduction hearing implant: indication criteria, surgery and a systematic review of the literature. Clin Otolaryngol..

[23] Hua H., Goossens T., Lewis A.T. (2022). Increased maximum power output may improve speech recognition with bone conduction hearing devices. Int J Audiol..

[24] Gerdes T., Salcher R.B., Schwab B., Lenarz T., Maier H. (2016). Comparison of audiological results between a transcutaneous and a percutaneous bone conduction instrument in conductive hearing loss. Otol Neurotol..

[25] Fan Y., Zhang Y., Wang P. (2014). The efficacy of unilateral bone-anchored hearing devices in Chinese Mandarin-speaking patients with bilateral aural atresia. JAMA Otolaryngol Head Neck Surg..

[26] Baker A., Fanelli D., Kanekar S., Isildak H. (2016). A review of temporal bone CT imaging with respect to pediatric bone-anchored hearing aid placement. Otol Neurotol..

[27] Charusripan P., Chinachatchawarat M., Rojvachiranonda N. (2025). Temporal bone thickness analysis in craniofacial anomalies: key considerations for bone conduction hearing implants. Eur Arch Otorhinolaryngol..

[28] Verhaegen V.J., Mylanus E.A., Cremers C.W., Snik A.F. (2008). Audiological application criteria for implantable hearing aid devices: a clinical experience at the Nijmegen ORL clinic. Laryngoscope..

[29] Rahne T., Plontke S.K. (2022). Systematic and audiological indication criteria for bone conduction devices and active middle ear implants. Hear Res..

[30] Gawliczek T., Wimmer W., Caversaccio M., Kompis M. (2021). Influence of compression thresholds and maximum power output on speech understanding with bone-anchored hearing systems. Biomed Res Int..

[31] Teunissen E.M., Aukema T.W., Banga R. (2024). Evaluation of clinical performance of ponto implantation using a minimally invasive surgical technique-a prospective multicenter study. Otol Neurotol..

[32] van Barneveld D.C.P.B., Kok H.J.W., Noten J.F.P., Bosman A.J., Snik A.F.M. (2018). Determining fitting ranges of various bone conduction hearing aids. Clin Otolaryngol..

[33] Håkansson B., Reinfeldt S., Persson A.C. (2019). The bone conduction implant - a review and 1-year follow-up. Int J Audiol..

[34] Kruyt I.J., Nelissen R.C., Mylanus E.A.M., Hol M.K.S. (2012). Assessment of more than 1,000 implanted percutaneous bone conduction devices: skin reactions and implant survival. Otol Neurotol..

[35] Dun C.A., Faber H.T., de Wolf M.J., Mylanus E.A., Cremers C.W., Hol M.K. (2012). Assessment of more than 1,000 implanted percutaneous bone conduction devices: skin reactions and implant survival. Otol Neurotol..

[36] Shapiro S., Ramadan J., Cassis A. (2018). BAHA skin complications in the pediatric population: systematic review with meta-analysis. Otol Neurotol..

[37] Sprinzl G., Toner J., Koitschev A. (2023). Multicentric study on surgical information and early safety and performance results with the Bonebridge BCI 602: an active transcutaneous bone conduction hearing implant. Eur Arch Otorhinolaryngol..

[38] Hodgetts W.E., Scollie S.D., Swain R. (2006). Effects of applied contact force and volume control setting on output force levels of the BAHA Softband. Int J Audiol..

[39] Di Berardino F., Ciavarro G., Fumagalli G. (2024). A non-surgical wearable option for bone conduction hearing implants: a comparative study with conventional bone conduction hearing aids mounted on eyeglasses. Audiol Res..

[40] Favoreel A., Heuninck E., Mansbach A.L. (2020). Audiological benefit and subjective satisfaction of children with the ADHEAR audio processor and adhesive adapter. Int J Pediatr Otorhinolaryngol..

[41] Kuthubutheen J., Broadbent C., Marino R., Távora-Vieira D. (2020). The use of a novel, nonsurgical bone conduction hearing aid system for the treatment of conductive hearing loss. Otol Neurotol..

[42] Zernotti M.E., Alvarado E., Zernotti M., Claveria N., Di Gregorio M.F. (2021). One-year follow-up in children with conductive hearing loss using ADHEAR. Audiol Neurootol..

[43] Dahm V., Auinger A.B., Liepins R., Baumgartner W.D., Riss D., Arnoldner C. (2019). A randomized cross-over trial comparing a pressure-free, adhesive to a conventional bone conduction hearing device. Otol Neurotol..

[44] Krijnen H.K., Sterry L., Child-Hymas A.E., Osborne M.S., Hol M., McDermott A.L. (2025). Audiological performance, complications, and compliance of the ADHEAR bone conduction device in a paediatric patient cohort. Audiol Neurootol.

[45] Kou T., Shen W., Li H.Z. (2025). Effectiveness of the SoundBite bone-conduction device in improving audibility, sound localization, and speech recognition for patients with single-sided deafness. Otol Neurotol..

[46] Kim G., Ju H.M., Lee S.H., Kim H.S., Kwon J.A., Seo Y.J. (2017). Efficacy of bone-anchored hearing aids in single-sided deafness: a systematic review. Otol Neurotol..

[47] Murray M., Miller R., Hujoel P., Popelka G.R. (2011). Long-term safety and benefit of a new intraoral device for single-sided deafness. Otol Neurotol..

[48] Tjellström A., Lindström J., Hallén O., Albrektsson T., Brånemark P.I. (1981). Osseointegrated titanium implants in the temporal bone. A clinical study on bone-anchored hearing aids. Am J Otol..

[49] Badran K., Arya A.K., Bunstone D., Mackinnon N. (2009). Long-term complications of bone-anchored hearing aids: a 14-year experience. J Laryngol Otol..

[50] Ellsperman S.E., Nairn E.M., Stucken E.Z. (2021). Review of bone conduction hearing devices. Audiol Res..

[51] Hao T., Ijaz A., Cho W.S., Kasbekar A. (2025). Percutaneous bone-anchored hearing rehabilitation in adults: the Nottingham experience over a five-year period. J Laryngol Otol..

[52] Hol M.K., Kunst S.J., Snik A.F., Bosman A.J., Mylanus E.A., Cremers C.W. (2010). Bone-anchored hearing aids in patients with acquired and congenital unilateral inner ear deafness (Baha CROS): clinical evaluation of 56 cases. Ann Otol Rhinol Laryngol..

[53] Bosman A.J., Hol M.K., Snik A.F., Mylanus E.A., Cremers C.W. (2003). Bone-anchored hearing aids in unilateral inner ear deafness. Acta Otolaryngol..

[54] Holgers K.M., Tjellström A., Bjursten L.M., Erlandsson B.E. (1988). Soft tissue reactions around percutaneous implants: a clinical study of soft tissue conditions around skin-penetrating titanium implants for bone-anchored hearing aids. Am J Otol..

[55] Lagerkvist H., Carvalho K., Holmberg M., Petersson U., Cremers C., Hultcrantz M. (2020). Ten years of experience with the Ponto bone-anchored hearing system-A systematic literature review. Clin Otolaryngol..

[56] Hultcrantz M. (2011). Outcome of the bone-anchored hearing aid procedure without skin thinning: a prospective clinical trial. Otol Neurotol..

[57] Caspers C.J.I., Kruyt I.J., Mylanus E.A.M., Hol M.K.S. (2020). Six-month clinical outcomes for bone-anchored hearing implants: comparison between minimally invasive ponto surgery and the linear incision technique with tissue preservation. Otol Neurotol..

[58] Strijbos R.M., Straatman L.V., Calon T.G.A. (2021). Long-term outcomes of the minimally invasive ponto surgery vs. linear incision technique with soft tissue preservation for installation of percutaneous bone conduction devices. Front Neurol..

[59] Bezdjian A., Bruijnzeel H., Daniel S.J., Grolman W., Thomeer H.G.X.M. (2017). Preliminary audiologic and peri-operative outcomes of the Sophono™ transcutaneous bone conduction device: A systematic review. Int J Pediatr Otorhinolaryngol..

[60] Baker S., Centric A., Chennupati S.K. (2015). Innovation in abutment-free bone-anchored hearing devices in children: Updated results and experience. Int J Pediatr Otorhinolaryngol..

[61] Magliulo G., Iannella G., De Vincentiis M. (2018). Transcutaneous bone conductive implants in patients with conductive/mixed hearing loss: audiological outcomes in noise condition. Acta Otolaryngol..

[62] Hol M.K., Nelissen R.C., Agterberg M.J., Cremers C.W., Snik A.F. (2013). Comparison between a new implantable transcutaneous bone conductor and percutaneous bone-conduction hearing implant. Otol Neurotol..

[63] Nelissen R.C., Agterberg M.J., Hol M.K., Snik A.F. (2016). Three-year experience with the Sophono in children with congenital conductive unilateral hearing loss: tolerability, audiometry, and sound localization compared to a bone-anchored hearing aid. Eur Arch Otorhinolaryngol..

[64] Rohani S.A., Bartling M.L., Ladak H.M., Agrawal S.K. (2020). The BONEBRIDGE active transcutaneous bone conduction implant: effects of location, lifts and screws on sound transmission. J Otolaryngol Head Neck Surg..

[65] Ghinelli F., Steeds C., Wells L. (2026). Cost-effectiveness Analysis Comparing Osia System to Percutaneous Bone Conduction Devices in Sweden. Otol Neurotol..

[66] Goldstein M.R., Bourn S., Jacob A. (2021). Early Osia® 2 bone conduction hearing implant experience: Nationwide controlled-market release data and single-center outcomes. Am J Otolaryngol..

[67] Stevens S.M., Meyer A., Rivas A. (2025). Outcomes Following Cochlear Osia 2 Implantation in Patients Ages 5-11 Years: A Multi-Center Trial. Laryngoscope..

[68] Key S., Mohamed N., Da Cruz M., Kong K., Hasan Z. (2023). Systematic review and meta-analysis of a new active transcutaneous bone conduction implant. Laryngoscope..

[69] Hol M.K.S., Aukema T.W., Ubbink S.W.J. (2025). A prospective, multicenter clinical investigation on the safety and performance of a new active transcutaneous bone-anchored implant system. Otol Neurotol..

[70] Kelley P.E., Scholes M.A. (2007). Microtia and congenital aural atresia. Otolaryngol Clin North Am..

[71] Acosta-Rodríguez A., Reza-López S.A., Aguilar-Torres C.R., Hinojos-Gallardo L.C., Chávez-Corral D.V. (2025). A systematic review of congenital external ear anomalies and their associated factors. Front Pediatr..

[72] Huang Y., Huang X., Li K., Yang Q. (2023). Risk factors of isolated microtia: a systematic review and meta-analysis. Plast Reconstr Surg..

[73] Casazza G., Meier J.D. (2017). Evaluation and management of syndromic congenital hearing loss. Curr Opin Otolaryngol Head Neck Surg..

[74] Yang T., Guo L., Wang L., Diagnosis Yu X. (2019). Intervention, and prevention of genetic hearing loss. Adv Exp Med Biol..

[75] Jahrsdoerfer R.A., Yeakley J.W., Aguilar E.A., Cole R.R., Gray L.C. (1992). Grading system for the selection of patients with congenital aural atresia. Am J Otol..

[76] Ahn J., Baek S.Y., Kim K., Cho Y.S. (2017). Predictive factors for hearing outcomes after canaloplasty in patients with congenital aural atresia. Otol Neurotol..

[77] Ahn J., Ryu G., Kang M., Cho Y.S. (2018). Long-term hearing outcome of canaloplasty with partial ossicular replacement in congenital aural atresia. Otol Neurotol..

[78] VARD Silva, Pauna H.F., Guimarães G.C., Lavinsky J., Linder T.E., Castilho A.M. (2025). Application of an active middle ear implant in congenital middle ear malformations: a contemporary review. Braz J Otorhinolaryngol..

[79] Zhang N., Li Y., Ma X. (2020). Isolated congenital middle ear malformations: comparison of preoperative high-resolution CT and surgical findings. Ann Otol Rhinol Laryngol..

[80] Ikeda A.K., Bhrany A.D., Sie K.C.Y., Bly R.A. (2021). Management of patients with unilateral microtia and aural atresia: recent advances and updates. Curr Opin Otolaryngol Head Neck Surg..

[81] Oliver E.R., Hughley B.B., Shonka D.C., Kesser B.W. (2011). Revision aural atresia surgery: indications and outcomes. Otol Neurotol..

[82] De la Cruz A., Teufert K.B. (2003). Congenital aural atresia surgery: long-term results. Otolaryngol Head Neck Surg..

[83] de Alarcon A., Choo D.I. (2007). Controversies in aural atresia repair. Curr Opin Otolaryngol Head Neck Surg..

[84] Verhaert N., Fuchsmann C., Tringali S., Lina-Granade G., Truy E. (2011). Strategies of active middle ear implants for hearing rehabilitation in congenital aural atresia. Otol Neurotol..

[85] Jiang C., Zhao C., Chen B. (2021). Auricular reconstruction using Medpor combined with different hearing rehabilitation approaches for microtia. Acta Otolaryngol..

[86] Bulstrode N.W., Jovic T.H. (2025). Advanced care for management of microtia using a two-stage autologous costal cartilage technique for auricular reconstruction: surgical method, outcomes, and future directions. J Craniofac Surg..

[87] Ronde E.M., Esposito M., Lin Y., van Etten-Jamaludin F.S., Bulstrode N.W., Breugem C.C. (2021). Long-term aesthetics, patient-reported outcomes, and auricular sensitivity after microtia reconstruction: a systematic review. J Plast Reconstr Aesthet Surg..

[88] Romo T., Morris L.G., Reitzen S.D., Ghossaini S.N., Wazen J.J., Kohan D. (2009). Reconstruction of congenital microtia-atresia: outcomes with the Medpor/bone-anchored hearing aid-approach. Ann Plast Surg..

[89] Wang Y., Xing W., Liu T. (2018). Simultaneous auricular reconstruction combined with bone bridge implantation-optimal surgical techniques in bilateral microtia with severe hearing impairment. Int J Pediatr Otorhinolaryngol..

[90] Ryan I.A., Tolley P.D., Han N.A. (2025). Concurrent one-stage polyethylene ear reconstruction and bone conduction hearing device placement: a novel technique and case series. Plast Reconstr Surg..

[91] Ghadersohi S., Haville S., Hedman M. (2021). Socioeconomic and clinical factors influencing treatment selection in microtia and aural atresia. Int J Pediatr Otorhinolaryngol..

[92] Harford E., Dodds E. (1966). The clinical application of CROS. A hearing aid for unilateral deafness. Arch Otolaryngol..

[93] Pedley A.J., Kitterick P.T. (2017). Contralateral routing of signals disrupts monaural level and spectral cues to sound localisation on the horizontal plane. Hear Res..

[94] Snapp H.A., Hoffer M.E., Liu X., Rajguru S.M. (2017). Effectiveness in rehabilitation of current wireless CROS technology in experienced bone-anchored implant users. Otol Neurotol..

[95] Prejban D.A., Hamzavi J.S., Arnoldner C. (2018). Single sided deaf cochlear implant users in the difficult listening situation: speech perception and subjective benefit. Otol Neurotol..

[96] Desmet J.B., Wouters K., De Bodt M., Van de Heyning P. (2012). Comparison of 2 implantable bone conduction devices in patients with single-sided deafness using a daily alternating method. Otol Neurotol..

[97] Peters J.P., Smit A.L., Stegeman I., Grolman W. (2015). Review: bone conduction devices and contralateral routing of sound systems in single-sided deafness. Laryngoscope..

[98] Choi J.E., Ma S.M., Park H., Cho Y.S., Hong S.H., Moon I.J. (2019). A comparison between wireless CROS/BiCROS and soft-band BAHA for patients with unilateral hearing loss. PLoS One..

[99] Peters J.P.M., van Heteren J.A.A., Wendrich A.W. (2021). Short-term outcomes of cochlear implantation for single-sided deafness compared to bone conduction devices and contralateral routing of sound hearing aids-Results of a Randomised controlled trial (CINGLE-trial). PLoS One..

[100] Wendrich A.W., van Heteren J.A.A., Peters J.P.M. (2023). Choice of treatment evaluated after trial periods with bone conduction devices and contralateral routing of sound systems in patients with single-sided deafness. Laryngoscope Investig Otolaryngol.

[101] Wendrich A.W., Kroese T.E., Peters J.P.M., Cattani G., Grolman W. (2017). Systematic review on the trial period for bone conduction devices in single-sided deafness: rates and reasons for rejection. Otol Neurotol..

[102] Akeroyd M.A. (2006). The psychoacoustics of binaural hearing. Int J Audiol.

[103] Kitterick P.T., Smith S.N., Lucas L. (2016). Hearing instruments for unilateral severe-to-profound sensorineural hearing loss in adults: a systematic review and meta-analysis. Ear Hear..

[104] Bagatto M., DesGeorges J., King A. (2019). Consensus practice parameter: audiological assessment and management of unilateral hearing loss in children. Int J Audiol..

[105] Gordon K., Henkin Y., Kral A. (2015). Asymmetric hearing during development: the aural preference syndrome and treatment options. Pediatrics..

[106] Park L.R., Griffin A.M., Sladen D.P., Neumann S., Young N.M. (2022). American cochlear implant alliance task force guidelines for clinical assessment and management of cochlear implantation in children with single-sided deafness. Ear Hear..

[107] Mertens G., Gilles A., Bouzegta R., Van de Heyning P. (2018). A prospective randomized crossover study in single sided deafness on the new non-invasive adhesive bone conduction hearing system. Otol Neurotol..

[108] Luo Q., Shen Y., Chen T. (2020). Effects of SoundBite bone conduction hearing aids on speech recognition and quality of life in patients with single-sided deafness. Neural Plast..

[109] Hampton T., Milinis K., Whitehall E., Sharma S. (2022). Association of bone conduction devices for single-sided sensorineural deafness with quality of life: a systematic review and meta-analysis. JAMA Otolaryngol Head Neck Surg..

[110] Saliba I., Nader M.E., El Fata F., Leroux T. (2011). Bone anchored hearing aid in single sided deafness: outcome in right-handed patients. Auris Nasus Larynx..

[111] Niparko J.K., Cox K.M., Lustig L.R. (2003). Comparison of the bone anchored hearing aid implantable hearing device with contralateral routing of offside signal amplification in the rehabilitation of unilateral deafness. Otol Neurotol..

[112] Holgers K.M., Håkansson B.E. (2002). Sound stimulation via bone conduction for tinnitus relief: a pilot study. Int J Audiol..

[113] Bahmad F., Cardoso C.C., Caldas F.F. (2019). Hearing rehabilitation through bone-conducted sound stimulation: preliminary results. Int Arch Otorhinolaryngol..

[114] Lekue A., Lassaletta L., Sánchez-Camón I., Pérez-Mora R., Gavilán J. (2013). [Quality of life in patients implanted with the BAHA device depending on the aetiology]. Acta Otorrinolaringol Esp..

[115] Wendrich A.W., Assouly K.K.S., van Heteren J.A.A. (2024). Tinnitus reduction in patients with single-sided deafness: the effect of cochlear implantation, bone conduction devices, and contralateral routing of sound hearing aids investigated in a randomized controlled trial. Front Neurol..

[116] Donato M., Santos R., Correia F., Escada P. (2021). Single-sided deafness: bone conduction devices or cochlear implantation? A systematic review with meta-analysis. Acta Otorrinolaringol Esp (Engl Ed).

[117] Tunkel D.E., Bauer C.A., Sun G.H. (2014). Clinical practice guideline: tinnitus. Otolaryngol Head Neck Surg..

[118] Buchman C.A., Roush P.A., Teagle H.F., Brown C.J., Zdanski C.J., Grose J.H. (2006). Auditory neuropathy characteristics in children with cochlear nerve deficiency. Ear Hear..

[119] Clemmens C.S., Guidi J., Caroff A. (2013). Unilateral cochlear nerve deficiency in children. Otolaryngol Head Neck Surg..

[120] Tahir E., Bajin M.D., Jafarov S. (2020). Inner-ear malformations as a cause of single-sided deafness. J Laryngol Otol..

[121] van Beeck Calkoen E.A., Sanchez Aliaga E., Merkus P. (2017). High prevalence of abnormalities on CT and MR imaging in children with unilateral sensorineural hearing loss irrespective of age or degree of hearing loss. Int J Pediatr Otorhinolaryngol..

[122] Lipschitz N., Kohlberg G.D., Scott M., Greinwald J.H. (2020). Imaging findings in pediatric single-sided deafness and asymmetric hearing loss. Laryngoscope..

[123] Nakano A., Arimoto Y., Matsunaga T. (2013). Cochlear nerve deficiency and associated clinical features in patients with bilateral and unilateral hearing loss. Otol Neurotol..

[124] Pollaers K., Thompson A., Kuthubutheen J. (2020). Cochlear nerve anomalies in paediatric single-sided deafness: prevalence and implications for cochlear implantation strategies. J Laryngol Otol..

[125] Casselman J.W., Offeciers F.E., Govaerts P.J. (1997). Aplasia and hypoplasia of the vestibulocochlear nerve: diagnosis with MR imaging. Radiology..

[126] Adunka O.F., Roush P.A., Teagle H.F. (2006). Internal auditory canal morphology in children with cochlear nerve deficiency. Otol Neurotol..

[127] Simons J.P., Mandell D.L., Arjmand E.M. (2006). Computed tomography and magnetic resonance imaging in pediatric unilateral and asymmetric sensorineural hearing loss. Arch Otolaryngol Head Neck Surg..

[128] Glastonbury C.M., Davidson H.C., Harnsberger H.R., Butler J., Kertesz T.R., Shelton C. (2002). Imaging findings of cochlear nerve deficiency. AJNR Am J Neuroradiol..

[129] Orzan E., Pizzamiglio G., Gregori M., Marchi R., Torelli L., Muzzi E. (2021). Correlation of cochlear aperture stenosis with cochlear nerve deficiency in congenital unilateral hearing loss and prognostic relevance for cochlear implantation. Sci Rep..

[130] Tahir E., Çınar B., Özkan H.B., Yaralı M., Böke B., Sennaroğlu L. (2020). Successful use of a cochlear implant in a patient with bony cochlear nerve canal atresia. J Int Adv Otol.

[131] Young N.M., Kim F.M., Ryan M.E., Tournis E., Yaras S. (2012). Pediatric cochlear implantation of children with eighth nerve deficiency. Int J Pediatr Otorhinolaryngol..

[132] Miyanohara I., Miyashita K., Takumi K., Nakajo M., Kurono Y. (2011). A case of cochlear nerve deficiency without profound sensorineural hearing loss. Otol Neurotol..

[133] Usami S.I., Kitoh R., Moteki H. (2017). Etiology of single-sided deafness and asymmetrical hearing loss. Acta Otolaryngol..

[134] Colletti L., Shannon R.V., Colletti V. (2014). The development of auditory perception in children after auditory brainstem implantation. Audiol Neurootol..

[135] Snapp H.A., Holt F.D., Liu X., Rajguru S.M. (2017). Comparison of speech-in-noise and localization benefits in unilateral hearing loss subjects using contralateral routing of signal hearing aids or bone-anchored implants. Otol Neurotol..

[136] Finbow J., Bance M., Aiken S., Gulliver M., Verge J., Caissie R. (2015). A comparison between wireless CROS and bone-anchored hearing devices for single-sided deafness: a pilot study. Otol Neurotol..

[137] Jakob T.F., Speck I., Rauch A.K. (2022). Bone-anchored hearing system, contralateral routing of signals hearing aid or cochlear implant: what is best in single-sided deafness?. Eur Arch Otorhinolaryngol..

[138] Monasta L., Ronfani L., Marchetti F. (2012). Burden of disease caused by otitis media: systematic review and global estimates. PLoS One..

[139] Amali A., Hosseinzadeh N., Samadi S., Nasiri S., Zebardast J. (2017). Sensorineural hearing loss in patients with chronic suppurative otitis media: Is there a significant correlation?. Electron Physician..

[140] Kotzias S.A., Seerig M.M., Mello M.F.P.C. (2020). Ossicular chain reconstruction in chronic otitis media: hearing results and analysis of prognostic factors. Braz J Otorhinolaryngol..

[141] Backous D., Choi B.Y., Jaramillo R. (2022). Hearing rehabilitation of patients with chronic otitis media: a discussion of current state of knowledge and research priorities. J Int Adv Otol..

[142] Lewis A., Vanaelst B., Hua H. (2021). Success rates in restoring hearing loss in patients with chronic otitis media: A systematic review. Laryngoscope Investig Otolaryngol..

[143] El-Sayed Y. (1998). Bone conduction impairment in uncomplicated chronic suppurative otitis media. Am J Otolaryngol..

[144] Yung M., Vowler S.L. (2006). Long-term results in ossiculoplasty: an analysis of prognostic factors. Otol Neurotol..

[145] Fadda G., Merlino F., Cavallo G. (2024). Factors influencing audiologic outcomes in ossiculoplasty for chronic otitis media: a prospective multicentre study. Acta Otorhinolaryngol Ital..

[146] Dornhoffer J.L. (1998). Hearing results with the Dornhoffer ossicular replacement prostheses. Laryngoscope..

[147] Yung M., Tono T., Olszewska E. (2017). EAONO/JOS Joint Consensus Statements on the Definitions, Classification and Staging of Middle Ear Cholesteatoma. J Int Adv Otol..

[148] Yu H., He Y., Ni Y., Wang Y., Lu N., Li H. (2013). PORP vs. TORP: a meta-analysis. Eur Arch Otorhinolaryngol..

[149] Jung D.J., Lee H.J., Hong J.S. (2021). Prediction of hearing outcomes in chronic otitis media patients underwent tympanoplasty using ossiculoplasty outcome parameter staging or middle ear risk indices. PLoS One..

[150] Kim H., Ha J., Choo O.S., Park H., Choung Y.H. (2023). Which is Better for Ossiculoplasty Following Tympanomastoidectomy: Polycel® or Titanium?. Ann Otol Rhinol Laryngol..

[151] Skarżyński P.H., Ratuszniak A., Obrycka A., Lorens A., Skarżyński H. (2025). The Ponto Bone-Anchored Hearing System - Benefits Following Radical Mastoidectomy. Med Sci Monit..

[152] Plontke S.K., Lenarz T., Toner J. (2026). The Bonebridge BCI 602 safety and performance 1 year post-implantation in adults and children: a multicentric post-market study. Otol Neurotol..

[153] Kurihara S., Nakamura T., Kubuki K. (2023). Hearing outcome and predictors after implanting bone conduction or middle ear implants in ears with refractory otitis media. J Clin Med..

[154] Ardıç F.N., Gülmez M., Okuyucu Ş (2025). Evaluation of bone-anchored hearing instruments in chronic otitis media: a multicenter prospective study. Turk Arch Otorhinolaryngol..

[155] Nassiri A.M., Messina S.A., Benson J.C. (2024). Magnetic resonance imaging artifact associated with transcutaneous bone conduction implants: cholesteatoma and vestibular schwannoma surveillance. Otolaryngol Head Neck Surg..

[156] Polo R., Del Mar Medina M., Arístegui M. (2016). Subtotal petrosectomy for cochlear implantation: lessons learned after 110 cases. Ann Otol Rhinol Laryngol..

[157] D’Angelo G., Donati G., Bacciu A., Guida M., Falcioni M. (2020). Subtotal petrosectomy and cochlear implantation. Acta Otorhinolaryngol Ital..

[158] Silva V.A.R., Pauna H.F., Lavinsky J. (2023). Brazilian Society of Otology task force - Otosclerosis: evaluation and treatment. Braz J Otorhinolaryngol..

[159] Vincent R., Sperling N.M., Oates J., Jindal M. (2006). Surgical findings and long-term hearing results in 3,050 stapedotomies for primary otosclerosis: a prospective study with the otology-neurotology database. Otol Neurotol.

[160] Van Dijk F.S., Sillence D.O. (2014). Osteogenesis imperfecta: clinical diagnosis, nomenclature and severity assessment. Am J Med Genet A..

[161] Garretsen A.J., Cremers C.W., Huygén P.L. (1997). Hearing loss (in nonoperated ears) in relation to age in osteogenesis imperfecta type I. Ann Otol Rhinol Laryngol..

[162] Ugarteburu M., Cardoso L., Richter C.P., Carriero A. (2022). Treatments for hearing loss in osteogenesis imperfecta: a systematic review and meta-analysis on their efficacy. Sci Rep..

[163] Kohan D., Ghossaini S.N. (2019). Osseointegrated auditory devices-transcutaneous: Sophono and Baha attract. Otolaryngol Clin North Am..

[164] Hernández S., Ospina J.C., Gutiérrez-Gómez E., Rodríguez-Ruiz M.T., Escobar J.L. (2021). Long term cutaneous complications related to bone conduction hearing implants. A retrospective study (2004-2018). Auris Nasus Larynx.

[165] Ray J., Wanees E., Dawoud M.M., Abu Elnaga H., Abdelhafez T.A. (2023). Evaluating the effectiveness of bone conduction hearing implants in rehabilitation of hearing loss. Eur Arch Otorhinolaryngol..

[166] Wazen J., Smith J., Daugherty J. (2019). Implantable auditory devices: financial considerations and office-based implantation. Otolaryngol Clin North Am..

[167] Nader M.E., Beadle B.M., Roberts D.B., Gidley P.W. (2016). Outcomes and complications of osseointegrated hearing aids in irradiated temporal bones. Laryngoscope..

[168] Sharon J.D., Khwaja S.S., Drescher A., Gay H., Chole R.A. (2014). Osteoradionecrosis of the temporal bone: a case series. Otol Neurotol..

[169] Lovin B.D., Hernandez M., Gidley P.W. (2025). The longitudinal impact of radiotherapy on osseointegrated hearing aid outcomes and complications. Laryngoscope..

[170] Chen S.Y., Mancuso D., Lalwani A.K. (2017). Skin necrosis after implantation with the BAHA attract: a case report and review of the literature. Otol Neurotol..

[171] Giannantonio S., Scorpecci A., Pacifico C., Marsella P. (2018). A functional and anatomical comparison between two passive transcutaneous bone conduction implants in children. Int J Pediatr Otorhinolaryngol..

[172] Utrilla C., Gavilán J., García-Raya P., Calvino M., Lassaletta L. (2021). MRI after Bonebridge implantation: a comparison of two implant generations. Eur Arch Otorhinolaryngol..

[173] Steinmetz C., Mader I., Arndt S., Aschendorff A., Laszig R., Hassepass F. (2014). MRI artefacts after Bonebridge implantation. Eur Arch Otorhinolaryngol..

[174] Abdalla I., Choi J.S., Struyk G., Adams M.E., Huang T.C. (2024). Magnetic Resonancy Imaging Safety in Active Osseointegrated (Osia®) and cochlear implants: new technology creating confusion. Laryngoscope..

[175] Lein A., Baumgartner W.D., Landegger L.D. (2024). A MAUDE database analysis on the new generation of active bone conduction hearing implants. Laryngoscope Investig Otolaryngol..

[176] Mohamad S., Khan I., Hey S.Y., Hussain S.S. (2016). A systematic review on skin complications of bone-anchored hearing aids in relation to surgical techniques. Eur Arch Otorhinolaryngol..

[177] Kiringoda R., Lustig L.R. (2013). A meta-analysis of the complications associated with osseointegrated hearing aids. Otol Neurotol..

[178] Mulvihill D., Kumar R., Muzaffar J. (2019). Inter-rater reliability and validity of holgers scores for the assessment of bone-anchored hearing implant images. Otol Neurotol..

[179] Calvo Bodnia N., Foghsgaard S., Nue Møller M., Cayé-Thomasen P. (2014). Long-term results of 185 consecutive osseointegrated hearing device implantations: a comparison among children, adults, and elderly. Otol Neurotol..

[180] Kruyt I.J., Bours M.R.W., Rovers M.M., Hol M.K.S., Rongen J. (2020). Economic evaluation of percutaneous titanium implants for bone conduction hearing: a cost-benefit analysis. Otol Neurotol..

[181] Gutierrez J.A., Shannon C.M., Nguyen S.A., Meyer T.A., Lambert P.R. (2024). Comparison of quality of life outcomes for percutaneous versus transcutaneous implantable hearing devices: a systematic review and meta-analysis. Otol Neurotol..

[182] Brinkman D., Hill R., Hone S., Kieran S. (2023). Bone-anchored hearing aids: Percutaneous versus transcutaneous attachments - a health economics comparison in paediatric patients. Int J Pediatr Otorhinolaryngol..

[183] Skarzynski P.H., Cywka K.B., Czaplicka E.A., Skarzynski H. (2025). The Bonebridge active bone conduction hearing implant: safety, effectiveness and outcomes based on 355 patients. Clin Otolaryngol..

[184] Carnevale C., Morales-Olavarría C., Til-Pérez G., Sarría-Echegaray P. (2023). Bonebridge. Eur Arch Otorhinolaryngol..

[185] Lorente-Piera J., Manrique-Huarte R., Patricio de Lima J., Huarte-Irujo A., Manrique M. (2024). Bone Conduction Implants: Comparative of Audiometric Results and Quality-of-Life Bonebridge® versus Osia®. Audiol Neurootol..

[186] Kim Y., Choe G., Oh H., Choi B.Y. (2023). A comparative study of audiological outcomes and compliance between the Osia system and other bone conduction hearing implants. Eur Arch Otorhinolaryngol..

[187] Crowson M.G., Tucci D.L. (2016). Mini review of the cost-effectiveness of unilateral osseointegrated implants in adults: possibly cost-effective for the correct indication. Audiol Neurootol..

